# Recent Developments in Nitric Oxide Donors and Delivery for Antimicrobial and Anti-Biofilm Applications

**DOI:** 10.3390/molecules27030674

**Published:** 2022-01-20

**Authors:** Wee Han Poh, Scott A. Rice

**Affiliations:** 1 Singapore Centre for Environmental Life Sciences Engineering, Nanyang Technological University, Singapore 637551, Singapore; rscott@ntu.edu.sg; 2 School of Biological Sciences, Nanyang Technological University, Singapore 637551, Singapore; 3 The iThree Institute, The University of Technology Sydney, Sydney, NSW 2007, Australia

**Keywords:** nitric oxide, nitric oxide donors, antibacterial, biofilm

## Abstract

The use of nitric oxide (NO) is emerging as a promising, novel approach for the treatment of antibiotic resistant bacteria and biofilm infections. Depending on the concentration, NO can induce biofilm dispersal, increase bacteria susceptibility to antibiotic treatment, and induce cell damage or cell death via the formation of reactive oxygen or reactive nitrogen species. The use of NO is, however, limited by its reactivity, which can affect NO delivery to its target site and result in off-target effects. To overcome these issues, and enable spatial or temporal control over NO release, various strategies for the design of NO-releasing materials, including the incorporation of photo-activable, charge-switchable, or bacteria-targeting groups, have been developed. Other strategies have focused on increased NO storage and delivery by encapsulation or conjugation of NO donors within a single polymeric framework. This review compiles recent developments in NO drugs and NO-releasing materials designed for applications in antimicrobial or anti-biofilm treatment and discusses limitations and variability in biological responses in response to the use of NO for bacterial eradiation.

## 1. Introduction

The development of antibiotic resistance, especially in ESKAPE pathogens (*Enterococcus faecium*, *Staphylococcus aureus*, *Klebsiella pneumoniae*, *Acinetobacter baumannii*, *Pseudomonas aeruginosa*, and *Enterobacter* species), is considered to be one of the most pressing global health threats by the WHO. Antibiotic resistance may arise due to spontaneous mutations, intrinsic resistance, and horizontal gene transfer [1]. In addition to genetic changes, biofilm formation is also a major mechanism of antibiotic tolerance. Biofilms are communities of bacterial cells that are attached to a surface, or in aggregates, and are surrounded by a matrix composed of extracellular polysaccharides (EPS), eDNA, or proteins. Such biofilms provide a physical barrier that hinders antibiotic penetration and act as a hotspot for gene transfer. In addition, cells within a biofilm display physiological heterogeneity, which affects growth state dependent adaptive resistance [2]. Biofilm formation is estimated to also account for up to 80% of chronic infections, which has significant economic costs and increases patient morbidity and mortality [3]. Beyond the healthcare setting, biofilm formation in industrial pipelines, wastewater treatment facilities, cooling towers, and water distribution systems, as well as on ship hulls is also a major problem [4,5]. These issues necessitate the need to develop new methods to eradicate both planktonic and biofilm. To this end, the use of nitric oxide (NO) is a viable approach as, depending on its dose, NO can directly kill planktonic bacteria or disperse biofilms. Dispersal of biofilms is considered advantageous as it can overcome biofilm-specific and intrinsic mechanisms of tolerance and may additionally prevent the development of resistance in the absence of direct killing and strong selective pressures. In addition, NO can potentiate antibiotic treatment, and the use of NO donors in conjunction with antibiotics has been shown to even eliminate drug resistant strains [6,7]

NO is a ubiquitous signaling molecule. In the human body, NO regulates vessel homeostasis, has roles in platelet activation, prevention of thrombosis, inflammatory processes, and wound healing processes [8,9]. Dysfunctions in NO production and regulation have also been associated with chronic lung infections, especially in cystic fibrosis (CF) patients, and in wound infections [10,11]. The use of NO as a treatment strategy may hence be disadvantageous due to tissue toxicity and other side effects, or may be an advantage by offering a simultaneous solution to both infection related diseases and modulating NO dysfunction. For example, NO may improve lung function [10,11,12]. NO-releasing wound dressing have also been shown to reduce bacterial counts at the wound site and improve subsequent wound healing due to extended NO release [13]. Many developments have been made in recent years to improve the delivery of NO to its targeted site and reduce cytotoxicity or off-target effects. In this review, we summarize the current state of the art with respect to NO-delivery or releasing small molecules or macromolecular scaffolds for antibacterial or antibiofilm treatment.

## 2. NO Production, Concentration, and Activity

### 2.1. NO Production, Concentration, and Activity

**Figure 1:**
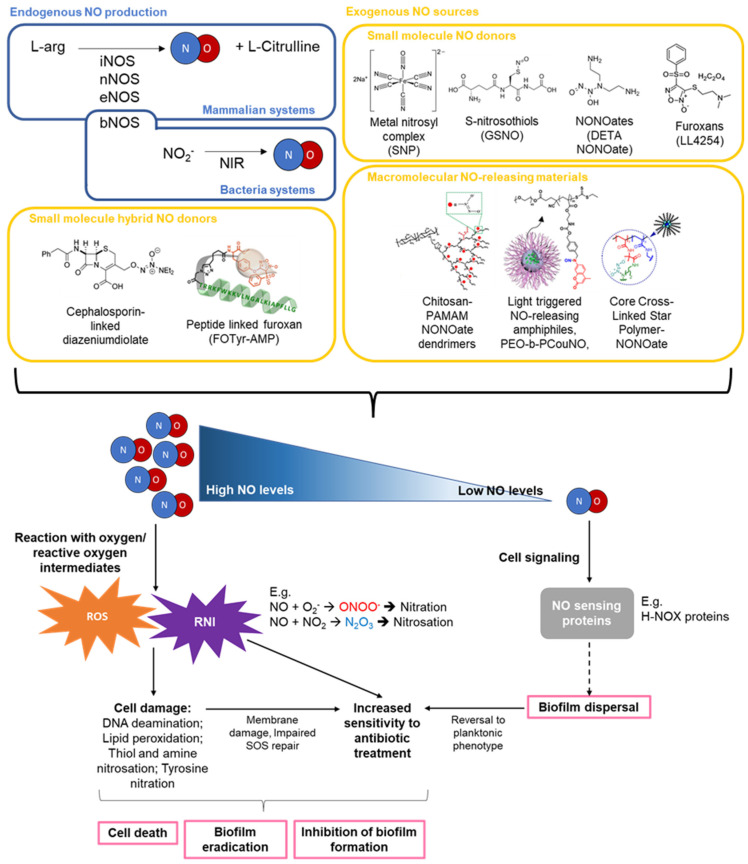
Examples of commonly used NO-donors, hybrid and macromolecular NO-releasing designs [21,22,23,24,25,26] NO at various concentrations can exert antimicrobial effects and/or potentiate the activity of antimicrobials.

In mammalian systems, NO is produced endogenously using L-arginine as a substrate at low concentrations by constitutively expressed NO synthases (NOS), such as neuronal NOS and endothelial NOS. In the presence of an immune stimuli, such as bacterial lipopolysaccharides, higher levels of NO can be generated by inducible NOS, which are expressed by both immune and some non-immune cells. Low levels of NO (<1 µM) are involved in signal transduction pathways and mediate physiological processes, while high levels of NO (>1 µM) are associated with cytotoxicity [14]. At high concentrations, NO can react with oxygen or other reactive oxygen intermediates (ROI) such as superoxide (O_2_^−^) to generate significant levels of reactive nitrogen species that can cause cell damage through free radical-mediated lipid peroxidation, thiol, and amine nitrosation, tyrosine nitration, and DNA deamination [15,16,17,18,19,20] (Figure 1).

Similar to mammalian systems, the activity of NO on bacteria and bacterial biofilms is dependent on its local concentration. In bacteria, NO may be generated from L-arginine by bacterial NOS [27,28,29]. Alternatively, NO may be produced through NOS-independent pathways, such as the reduction in nitrite to NO by nitrite reductases in denitrifying bacteria [30]. In general, at low concentrations (pM–µM), NO can mediate biofilm formation or disperse biofilms, while at higher concentrations (>1 mM), NO exerts a bactericidal effect in a similar manner as described above for mammalian cells. The effects of targeted or spontaneous release of high and low doses of NO can be found in Table 1.

### 2.2. NO Mediated Biofilm Formation and Dispersal

NO was initially found to be involved in biofilm formation and dispersal of *P. aeruginosa* [6]. Low concentrations of SNP (25 nM–2.5 mM), corresponding to approximately a thousand times lower levels of NO (25 pM to 2.5 μM NO) inhibited biofilm formation. At concentrations above 2.5 mM SNP (>2.5 μM NO), NO instead promoted biofilm formation. The effect of NO on biofilm inhibition and dispersal was later expanded to other Gram-negative bacteria such as *Serratia marcescens*, *Vibrio cholerae*, *E. coli*, *Fusobacterium nucleatum*, *Bacillus licheniformis*, and *Staphylococcus epidermis*, as well as clinical and multi-drug resistant isolates and even mixed species biofilms from water distribution and treatment systems. In these studies, NO donors like sodium nitroprusside (SNP), *S*-nitroso-*N*-acetylpenicillamine (SNAP), *S*-Nitrosoglutathione (GSNO), Proli NONOate, and NO releasing nanoparticles were found to be effective in reducing biofilms over a range of concentrations (nM–μM) [31,32].

### 2.3. Bacterial Species, Strains, Growth Conditions, and Stage of Biofilm Development Influence Bacterial Response to NO and the Effectiveness of NO Treatment

The response of bacteria to NO is dependent on the bacterial strain and growth conditions. For example, unlike previous examples where biofilm formation was inhibited at low NO concentrations and promoted at high NO levels, *S. aureus* displayed increases in biofilm biomass when exposed to 0.9–2 µM of NONOate, while biofilms were reduced at 125–1000 µM of NONOate [33,34]. Interestingly, low NO levels (0.7–2.3 µM) corresponded with rhinosinusitis while normal sinonasal NO levels are approximately 200–830 µM [34]. Differences in sensitivity towards the antimicrobial activity of NO may also be due to differences in molecular weight thiols and other innate resistance and oxidative and nitrosative stress responses in different species [35,36,37,38]. Even for the same strain, NO may have different effects depending on the type of infection. In a study using *Streptococcus pneumoniae*, NO had no effect on the survival of mice in a pneumococcal pneumonia model of infection when the bacteria were administered intranasally, but it was required for local anti-bacterial activity in the lungs. However, during systemic bacteremia, where bacteria were administered intravenously, NO was associated with increased bacterial load and reduced survival [39].

Anaerobic or aerobic conditions may also influence the activity of NO donors. For example, twofold higher concentrations of NO-releasing chitosan oligosaccharides (COS-NO) and NO levels were needed for bactericidal effects on non-growing, planktonic mucoid *P. aeruginosa* strains compared to non-mucoid strains [40]. Subsequently, it was found that higher concentrations of COS-NO, but not significantly different NO concentrations, were needed to eradicate actively growing planktonic mucoid and non-mucoid *P. aeruginosa* under aerobic conditions. This effect is likely due to the consumption of NO by oxygen under aerobic conditions. Within biofilms, lower COS-NO and NO doses could eradicate both mucoid and non-mucoid *P. aeruginosa* under anaerobic conditions compared to aerobic conditions. In contrast to COS-NO, the anti-biofilm efficacy of hyperbranched NO-releasing polymers against multi-species dental biofilms were reduced under anaerobic conditions [41]. The authors hypothesized that the bacteria, being anaerobes, were more susceptible to treatments under aerobic conditions.

In addition to growth conditions, the treatment regime may also play a role in the bacterial response to NO. Barnes et al. first showed that repeated treatment of *P. aeruginosa* biofilms with the NO-donor MAHMA NONOate at micromolar concentrations had limited additional effects on biofilm dispersal; although, NO may continue to play an inhibitory role on biofilm growth compared to untreated samples [42]. In addition, older *P. aeruginosa* biofilms were less susceptible to treatment by MAHMA NONOate than early stage biofilms [42]. Zhu et al. showed that *P. aeruginosa* biofilms that were pre-treated with 10 µM of the NO-donor spermine NONOate were resistant to subsequent dispersal by 100 µM of the NO donor (~4 µM NO) due to upregulation of *fhp*, which has NO-scavenging activity [43]. Imidazole, which can inhibit Fhp, attenuated this effect, but only in mature (19 h old) biofilms and not younger biofilms (6 h). Alternatively, 6 h biofilms could be dispersed by increasing the concentration of NO donor used to 200–500 µM, possibly by overwhelming NO scavenging mechanisms [43].

### 2.4. Combinations of NO and Antibiotic Treatments

In general, NO can improve the killing of both biofilm and planktonic *P. aeruginosa*, *Listeria monocytogenes*, *S. aureus, B. cepacia*, and other drug-resistant strains when used with various classes of antibiotics, such as macrolides (azithromycin), fluoroquinolone (sparfloxacin, ciprofloxacin, and ofloxacin), *β*-lactam drugs (aztreonam), cephalosporins (ceftazidime), polycationic peptides (colistin), and aminoglycosides (tobramycin), as well as other antimicrobial agents such as gallium (III) and antimicrobial peptides [32,44,45,46,47,48]. NO mediated potentiation of antibiotics was also observed in *P. aeruginosa*, where inactivation of nitrite-dependent NO synthesis promoted development of antibiotic resistance against cefoperazone–sulbactam [37].

At low doses of NO, the potentiation of antibiotic efficacy may be due to active dispersal of the biofilm, which reverses the tolerance in biofilm cells, which once again become as susceptible as planktonic cells [44,49]. However, NO can also increase susceptibility towards antibiotic treatment in the absence of biofilm dispersal events [50]. It is possible that under such conditions, reactive oxygen species (ROS) and reactive nitrogen intermediates (RNI) produced after NO generation cause membrane damage and improve antibiotic uptake and bacteria susceptibility [47]. Alternatively, ROS and RNI, via impairment of SOS repair mechanisms, can further exacerbate DNA damage caused by interference of fluoroquinolones antibiotics on DNA replication [48]. NO may also drive selection, as in the case of *Mycobacterium abscessus*, for a more azithromycin susceptible morphotype [51].

Despite the efficacy of NO in killing and potentiating antibiotic activity, conflicting reports of NO on aminoglycoside activity have been reported. Aminoglycosides are taken up through active electron transport and, thus, inherently lack activity against anaerobic bacteria [52]. In this manner, NO can also protect *P. aeruginosa* and *S. aureus* against aminoglycosides via NO-mediated repression of respiration and subsequent reduction in energy-dependent drug uptake [53]. However, NO, in combination with tobramycin, can also improve cell and biofilm eradication [6,32,45]. In some cases, however, synergism of the two drugs depends on their administration. In tobramycin resistant *P. aeruginosa* expressing aminoglycoside modification enzymes, simultaneous administration of NO-releasing chitosan (COS/NO) with tobramycin does not further improve antibiotic activity [47]. However, pre-treatment of biofilms of tobramycin resistant strains with NO did improve antibiotic efficacy [47]. The benefits of NO-pretreatment is supported by Huang et al., where longer NO pre-treatment times of >4 h further improved ofloxacin efficacy of biofilm cells to a similar extent as susceptible planktonic cells [49].

Due to the beneficial effect of both NO and antibiotic treatment, NO has been incorporated into a variety of delivery platforms. Nguyen et al. attached a NO-releasing moiety to gentamicin within a polymeric nanoparticle for simultaneous release of both compounds and observed synergistic effects against *P. aeruginosa* [54]. In addition, Duan et al. designed photoreceptive of NO-releasing amphiphiles that can self-assemble into vesicles and incorporate the water-soluble gentamicin antibiotic within the vesicle, enabling sequential release of NO and gentamicin upon photoirradiation [55].

## 3. Gaseous NO and Other Low Molecular Weight (LMW) NO Donors

NO can be administered directly through NO gas inhalation or via low molecular weight NO donors or polymeric NO delivery platforms. The following sections, as well as Table 2, would summarize some common classes of NO donors often used in NO studies as well as newer developments in small molecule NO donors.

### 3.1. Gaseous NO

NO gas has long been administered for pulmonary dilation and treatment of pulmonary hypertension [77,78]. More recently, NO gas has been explored as an antimicrobial and antibiofilm treatment against both lung infections, especially chronic infections present in cystic fibrosis patients, and wound infections [32,59,60,61,62,79,80,81,82,83].

One of the main side effects of the application of gaseous NO, when administered as inhaled NO for the treatment of lung infection, is the formation of the irritant NO_2_ and methemoglobin (MetHb). In the latter, NO oxidizes heme to its ferric state and reduces the ability of hemoglobin to transport oxygen. As such, one of the main concerns of inhaled NO treatment is to keep MetHb and NO_2_ levels within tolerable levels. Fortunately, the side effects of inhaled NO can be easily reversed by discontinuation of the gas. Depending on the study, breathing NO at <40 ppm, up to 80 ppm or up to 128 vpm for 3 h does not significantly increase the formation of MetHb [78,84,85]. At 160 ppm, MetHb levels reached 3.9% within 2 h, below the tolerable range of <5% [86]. Hence, clinical studies involving the use of high doses of inhaled NO for antimicrobial purposes often make use of intermittent administration of high doses of NO [67].

Initial studies indicated that intermittent exposure to 160 ppm NO in vitro could reduce the numbers of clinical multidrug resistant (MDR) *S. aureus* and *E. coli* strains, as well as antibiotic-resistant *P. aeruginosa* by over 5-log with no host cellular toxicity observed, MetHb < 2.5% and NO_2_ < 2 ppm [87]. NO administered intermittently at 160 ppm was well tolerated in multiple clinical trials or case studies involving chronic lung infections in cystic fibrosis patients [59,60,61]. In one study, this treatment regime reduced bacterial and fungal counts and improved patient lung function with NO_2_ averaging 4.0 ppm and MetHb < 3% during treatment [60]. In another study, 160 ppm NO reduced pulmonary colonization of antibiotic resistant *Burkholderia multivorans* in a 16-year-old CF patient. NO was administered with a mechanical ventilator fitted with NO_2_ scavenging chambers to reduce formation of the latter [61]. Lastly, intermittent dose at 160–240 ppm NO was used in a pilot study and in a compassion-use case for the treatment of pulmonary *M. abscessus* [58,59]. In both cases, the treatment was well tolerated. Different strains of *M. abscessus* of the latter study showed varying susceptibility to NO treatment in vitro, with the patient’s isolate being the least susceptible [58]. As such, *M. abscessus* was not fully eradicated from the lung infection. However, improvements in lung function and other selected quality of life markers were improved [58]. Further treatment improvements may be possible using co-treatment with antibiotics, as high doses of NO and amikacin and clofazimine have synergistic effects against *M. abscessus* in vitro [83].

High levels of NO have also been studied in murine models of infection, where NO inhalation (300 ppm for 12 min every 3 h for 48 h) reduced viable *K. pneumoniae* and MDR *Klebsiella* in lung tissues and improved survival rates [56]. In addition, 200 ppm NO gas was continuously administered in porcine normothermic ex vivo lung perfusion experiments with no significant differences in inflammatory cytokines compared to control groups and a NO_2_ level <2.8 ppm during treatment. In vitro, the gaseous NO reduced the numbers of all strains of *P. aeruginosa*, *S. aureus*, *E. coli*, and *Burkholderia cepacia* by about 4 logs. As such, taken together, continuous high doses of gaseous NO was recommended for use to reduce microbial infection in donor lungs during lung transplantation [57]. In contrast to high doses of NO, Howlin et al. used of a low dose of NO (10 ppm) inhalation over 7 d that significantly reduced *P. aeruginosa* biofilm aggregates in 12 CF patients, which may have potential in overcoming biofilm-associated antibiotic tolerance in biofilm associated infections with further reductions in the side effects of inhaled NO administration [32].

Gaseous NO has also been explored for treatment against skin infections [62,79]. Ghaffari et al. evaluated the use of gaseous NO against various pathogens and its effect on human dermal fibroblasts in culture [62]. In a continuous horizontal flow delivery system, 160 ppm NO over 24 h reduced *P. aeruginosa* and *S. aureus* bacterial growth by 90%. At 200 ppm, NO applied over an average of 4.1 h completely stopped bacteria growth of seven clinical strains, including isolates of *S. aureus*, *E. coli*, *P. aeruginosa*, and Group B *Streptococcus*. In addition, the dose of NO was non-cytotoxic over 48 h of exposure. In a follow up study, 200 ppm gaseous NO was applied for 8 h/day for 3 d on full thickness wounds infected with *S. aureus* [79]. Consistent with previous in vitro studies, gaseous NO significantly reduced wound bacterial content in in vivo models. In vitro, gaseous NO does not appear to interfere with re-epithelialization and angiogenesis during wound healing, further indicating its potential for treatment of chronic ulcers or wounds.

### 3.2. Metal Nitrosyl Complexes

Metal nitrosyl complexes consists of nitric oxide bonded to a transition metal. One representative NO donor of this class of compound is SNP, which has a long history of use clinically for the treatment of high blood pressure and cardiovascular diseases. SNP use has been shown to inhibit biofilm formation as well as induce biofilm dispersal and potentiate antibiotic or antimicrobial treatment in Gram-negative bacteria including *P. aeruginosa*, *V. cholerae*, and *E. coli* and the Gram-positive bacterium, *S. epidermidis* [6,63]. In *S. pneumoniae*, SNP (nM–mM) did not have effect on dispersal but reduced cell viability at higher concentrations (mM) [50]. One limitation of the use of SNP is the possibility of cyanide toxicity with prolong treatment [88]. As such, there is interest in using other classes of NO donors, such as *S*-nitrosothiols, which can be produced endogenously and have biological roles in mediating NO signalling via protein *S*-nitrosylation, and diazeniumdiolates (NONOates), which have well-defined NO release kinetics.

### 3.3. S-Nitrosothiols

*S*-nitrosothiols, such as GSNO and SNAP are generally referred to as RSNO compounds, where R denotes an organic group. *S*-nitrosothiols, such as GSNO, are found endogenously in mammalian systems, have a well-studied metabolism within the body, and have intrinsically low toxicity, making them attractive NO donors for therapeutic applications [89,90,91]. NO release from *S*-nitrosothiols can be spontaneous and can be modulated by the presence of transition-metal ions, particularly Cu^2+^, acids, or light irradiation [92,93,94,95,96]. Light based activation is particularly useful for spatial or temporal control of NO release. Low concentrations (1 µM) of GSNO and SNAP can prevent initial biofilm formation in *P. aeruginosa* in vitro [6]. At higher concentrations (mM), GSNO and *S*-nitroso-*N*-acetylcysteine (SNAC) were also inhibitory and bactericidal against bacterial isolates from keratitis infections [66]. In addition, RSNO can be readily incorporated into a polymeric macromolecular scaffold. Within such systems, only NO is released from the scaffold upon S-N bond cleavage, hence enabling localized NO release for antibacterial or anti-biofilm purposes [91].

### 3.4. N-Diazeniumdiolates

Diazeniumdiolates (NONOates) are compounds containing a diolate group −[N(O-)N=O] linked to another atom or compound (**X**). Depending on the identity of **X**, such as in the case of some C-bound diazeniumdiolates, NO may or may not be released from the NONOate [97]. In general, NO release from amine *N*-diazeniumdiolates is spontaneous under physiological conditions and follow first order rates, with theoretically 2 mol of NO released per mole of NONOate [97,98]. While not used clinically, NONOates are often used in research as a broad range of NONOates with different, but reproducible NO release half-lives and steady states are available, enabling researchers to evaluate the effects of short- and long-term exposure to NO [98]. Furthermore, diolate groups can be easily loaded onto amine moieties in polymeric materials for NO release, where NO release enhances bacteria and biofilm eradication [40,99,100,101,102,103,104,105]. NONOates have been used in multiple studies on NO-induced biofilm dispersal. Of which, MAHMA NONOate, and PROLI NONOate have been shown to reduce single and mixed species biofilms of bacteria from industrial membrane reactors and reverse osmosis membranes [42,67], while four NONOates showed varying activities against *Salmonella enterica* and *E. coli* O157:H7 biofilms [68]. In *P. aeruginosa*, spermine NONOate has been used in dispersal studies investigating resistance response to dispersal following NO pretreatment and the relation between NO response and iron signalling [43,106]. Separately, spermine NONOate was found to induce the best biofilm dispersal response against CF isolates compared to other NONOates, including MAHMA, PROLI, and DEA NONOate, which were evaluated [107].

### 3.5. Furoxans

Furoxans or furoxan derivatives are thermally stable, heterocyclic NO-donor compounds consisting of a 1,2,5-oxadiazole 2-oxide ring. NO release from furoxans can be spontaneous or thiol activated with substituent groups at positions 3 and 4 of the furoxan ring influencing the NO release kinetics [108,109]. Furoxans with fast NO-release kinetics can function as biofilm dispersal agents in a NO-dependent manner, whereas furoxans with slower or low NO-release had varying effects on inhibiting *P. aeruginosa* planktonic growth and/or biofilm formation, as well as pyoverdine production, in an NO-independent manner [69,70]. Such dual effects exerted by furoxans in turn make them interesting as potential new drugs.

### 3.6. Photo Responsive/Photoactivated Ruthenium Compounds

In the case of smaller, light-triggered NO-releasing molecules, Boce et al. designed a light responsive NO donor using ruthenium (Ru) nitrosyl complexes, which have low toxicity, good stability, and can release NO under light irradiation [65,110,111]. The ruthenium nitrosyl complex was efficient in NO photo-delivery upon one-photon absorption at 405 nm. However, it can also respond to two-photon absorption in the NIR region. The latter of which confers advantages of low toxicity, high selectivity, and deep penetration into biological tissues. Co-treatment of Ru-nitrosyl complexes with methicillin increased methicillin resistant *S. epidermidis* biofilm sensitivity to the antibiotic by 100-fold, suggesting that it may be a promising approach for biofilm eradication. In a similar fashion, Zhao et al. designed a boronic acid decorated Ru(II) compound with a NO-releasable group (RBNO) that releases NO through photo-induced N-nitrosamine bond cleavage [64]. The boronic acid group acts as a positively charged metal group, facilitating adhesion of the RBNO onto bacterial peptidoglycan or LPS via boron-polyol-based boronolectin chemistry. This confers selectivity in a bacteria–mammalian cell co-culture, eradicating *P. aeruginosa* while having negligible toxicity to the WI-38 cell line tested.

### 3.7. Hybrid-NO Donors

Hybrid NO compounds can be designed to improve the specificity of NO-release from small molecule NO donors, or for co-delivery of antimicrobials and NO for synergistic effects [72,76]. A series of cephalosporin-linked diazeniumdiolate NO-donor prodrugs (C3D) has been designed and studied for their antimicrobial and antibiofilm effects [25,71,72,73,74,75]. These prodrugs consist of a cephalosporin, a class of antibiotics containing a *β*-lactam ring, linked to a diazeniumdiolate NO donor. The hybrid compound is stable, and NO is only released from the diazeniumdiolate at the bacteria infection site upon cleavage of the *β*-lactam ring by bacterial produced *β*-lactamases, transpeptidases, or penicillin binding proteins (PBPs) [71,72].

First generation C3Ds were effective at dispersing in vitro *P. aeruginosa* biofilms grown for 6–44 h with or without prior induction of *β*-lactamase production by using sub-inhibitory concentrations of the *β*-lactam antibiotic, imipenem [72,75]. The prodrug did not show any antimicrobial effects, and additional antibiotics, such as ciprofloxacin, tobramycin, or colistin, must be administered to enhance the eradication of biofilm cells and for complete eradication of dispersed and planktonic cells [72,75]. Later generations of C3Ds have both direct antibacterial activity as well as NO-releasing capabilities [71,73]. In studies carried out on *S. pneumoniae* using PYRRO-C3D, NO-release from the prodrug was mediated by the non-*β*-lactamases producing bacteria. However, NO does not play a direct role in the anti-pneumococcal effects of the compound [71]. Instead, the compound had potent *β*-lactam-mediated antibacterial activity (MIC = 900 nM) and displayed higher potency than the amoxicillin and azithromycin against biofilms, suggesting that there was a synergistic effect when using the hybrid NO donor [71]. In further studies on non-typeable *Haemophilus influenzae* (NTHi) biofilms, PYRRO-C3D increased the susceptibility of NTHi biofilms grown in vitro on polystyrene surfaces or on primary respiratory epithelia towards azithromycin, presumably by NO-mediated modulation of the metabolic activity of the bacteria [73]. DEA NONOate was unable to elicit the same response, indicating that the targeted, slow but sustained NO release from PYRRO-C3D may be more beneficial for reducing biofilm associated antibiotic resistance than spontaneous NO donors [73]. Further developments subsequently yielded C3Ds with higher potency than the parental cephalosporin, ceftazidime, with improved *β*-lactamase stability and higher PBP reactivity for broad-spectrum antibacterial activity [75]. The new C3D had activity against ceftazidime-resistant *P. aeruginosa* in vitro biofilms and had efficacy in murine *P. aeruginosa* respiratory infection model.

Beyond C3Ds, antimicrobial peptides have also been conjugated to a NO-donating furoxan moiety. The hybrid compound displayed synergistic activity against *S. aureus* and *E. coli* in inhibition of biofilm growth, biofilm dispersal, and eradication of both biofilm and planktonic cells compared to the AMPs or furoxan donors alone, with low hemolysis and effects on blood pressure when administered subcutaneously in mice models [26].

In addition to linking an antibiotic to a NO donor, NO donors have also been combined with quorum sensing (QS) inhibitors to target different stages and pathways of biofilm development [76,112]. Fimbrolide QS inhibitors combined with nitrooxy or diazeniumdiolate NO donor groups had similar or improved biofilm inhibition activity against *P. aeruginosa* compared to their natural fimbrolide and parent compound or SNP [76]. Similarly, surface coatings consisting of dihydropyrrolones QS inhibitors linked to *N*-diazeniumdiolates further reduced colonization by *S. aureus* and *P. aeruginosa* through a non-bactericidal mechanism compared to either compound alone [112].

## 4. Macromolecular NO Donor Scaffolds

The clinical use of low molecular weight (LMW) NO donors may be limited by their stability under physiological conditions and limited NO loading. This can lead to a burst release that may result in off-target effects and cytotoxicity. Due to off-target NO release, LMW NO donors may also be unable to deliver an appropriate therapeutic dose to the targeted site over a desired duration of treatment. To overcome these limitations, NO delivery via macromolecular carriers offers a strategy for targeted, controlled, and sustained delivery of NO to the targeted site. NO donors can be loaded on, or attached to, a variety of materials, including inorganic, natural, and synthetic polymers, to fabricate NO-releasing nanoparticles (NPs), hydrogels, surfaces, and coatings. These macromolecular carriers offer several advantages: (1) improved NO stability, with tunable and favorable NO release kinetics as compared to LMW NO donors; (2) high NO loading due to increased sites for functionalization or increased surface area to volume ratio of nanomaterials; (3) controlled NO release in response to a stimulus. e.g., by light irradiation or pH changes; and (4) ability to combine with other compounds for synergistic effects, such as the incorporation of antibiotics within NO-releasing nanoparticles for treatment of bacterial infections. Ideally, the carrier would be multifunctional to achieve several of the advantageous features within a single framework. The subsequent sections, and the corresponding Table 3, will present various macromolecular NO-releasing platforms and their bactericidal activity, or ability to prevent of biofilm formation and/or disruption or dispersal of formed biofilms. A comparison of the pros and cons of different NO-releasing materials can be found in Table 4.

### General Properties of Macromolecular NO Donor Scaffolds That Can Influence Their Activity

The physical and surface properties of nanoparticles and macromolecular scaffolds can influence their activity independent of NO release. These include the size and shape of nanoparticles, which can affect biofilm penetration and particle–bacteria association. As bacterial cell membranes carry a net negative charge, scaffolds functionalized with positively charged groups, such as quaternary ammonium, can also better associate with the bacterial membrane, leading to cell death through membrane disruption. In some cases, NO-release and overall activity of the material may also be influenced by these physical properties. For example, NO release and biofilm dispersal activity by NONOate-functionalized POEGMA-b-PGMA NPs was dependent on whether it has a small spherical morphology or a longer worm-like morphology [129]. In dendrimers, NO release increased cytotoxicity against HGF-1 cell lines in propyl-functionalized dendrimers but reduced cytotoxicity for quaternary ammonium-modified dendrimers [100,102]. Such interactions would be discussed further within each of the later sections.

## 5. Natural Polymer-Based NO-Releasing Scaffolds

### 5.1. Types of Natural Polymer-Based NO-Releasing Scaffolds

Natural polymers such as chitosan and alginate are attractive as NO donor scaffolds due to their biodegradability and biocompatibility, and in the case of chitosan, innate antimicrobial activity [152,153]. The hydroxyl and carboxylic acid functional groups on the alginate backbone and primary amine groups on chitosan offer straightforward means of chemical modification to introduce NO donor groups, or addition of antimicrobial functionalities for synergistic antibacterial effects with NO treatment [21,92,113,114,118,119,120,154]. Cyclodextrins (CDs) have also been developed as tunable NO-releasing agents [121,122]. These are a family of cyclic oligosaccharides with a hydrophobic central cavity and hydrophilic exterior and could offer an advantage for delivery of hydrophobic drugs or antibiotics for bacterial control.

### 5.2. NO-Releasing Chitosan Oligosaccharides (COS/NO)

Early studies of NO-releasing chitosan scaffolds primarily focused on chitosan oligosaccharides (COS) and this class of compounds has been reviewed in part by Yang et al. [155]. Compared to high molecular weight (MW) chitosan, COS are more water-soluble at the basic pH required for diazeniumdiolate formation or thiol modification, and hence have improved NO loading [92,113]. In general, these COS/NO molecules displayed good antibacterial and anti-biofilm activity, reducing the viability of planktonic or biofilm *P. aeruginosa*, *E. coli*, and *S. epidermidis*, or their adherence to surfaces, with low cytotoxicity against the cell lines used [40,92,113,114,115].

### 5.3. Positive Charge of COS and Association of COS/NO with Bacterial Membranes or Biofilms the Main Driver of Antimicrobial Activity

The activity of COS/NO is influenced by both NO flux and association with bacterial membranes; although, the latter appears to have a more important role. For example, the primary thiol-modified chitosan-TBA-NO (total NO = 74 nmol/mg, maximum NO flux = 54 ppb/mg), which has lower NO storage and release than the tertiary thiol-modified chitosan-NAP-NO (total NO = 78 nmol/mg, maximum NO flux = 87 ppb/mg), was more effective against *P. aeruginosa* because the positively charged amine on the TBA unit promotes its association with the bacteria [92]. Similarly, PEGylation of the primary amines of chitosan 2/NO-5k gave rise to chitosan 3/5k, which has higher NO flux, but lower bactericidal activity, due to shielding of the amine moieties by PEGylation and reduced charge facilitated interaction with the bacteria. A higher concentration and greater NO dose of Chitosan 3/NO-5k (NO dose = 0.45 μmol/mL) was required for the bactericidal effects compared to Chitosan 2/NO-5k (NO dose = 0.10 μmol/mL) [113]. 

Within biofilms, NO and chitosan scaffolds can exert opposing effects. The cationic chitosan scaffold can increase cohesion of negatively charged *P. aeruginosa* biofilms and improve biofilm elasticity. In contrast, NO promotes biofilm disruption and dispersal. Nevertheless, NO-releasing COS have been found to physically disrupt *P. aeruginosa* biofilms and damage the biofilm matrix associated eDNA when used at concentrations above the MBEC [92,113,114]. As such, increasing the NO payload may minimize any underlying positive effects of the chitosan scaffold. Likewise, weakly mucoadhesive, negatively-charged modified COS had no discernible effects on CF sputum architecture due ineffective COS penetration into mucin matrix following negative-charge modification [156].

### 5.4. Chitosan Gels

Chitosan gels with encapsulated NO donors, such as isosorbide mononitrate (ISMN) or GNSO, with or without further encapsulation of antibiotics, also showed sustained NO release over 72 h and was highly effective in reducing the viability and preventing biofilm formation of *P. aeruginosa* and *S. aureus* in vitro and in vivo [13,116,118]. NO release from these chitosan gels is influenced by their hydration state and more NO is released in response to higher amounts of wound exudates at the site of infection [13]. In vitro, ~170.2 nmol NO/mg of NO-releasing chitosan (CS/NO) film was released with the addition of 500 µL of simulated wound fluid (SWF). This is ~10% more total NO released by 72 h compared to CS/NO films treated with 120 µL of SWF. The CS/NO film significantly decreased MRSA viability by more than 3 log in vitro, reduced biofilm biomass and improved wound healing by approximately seven times that of non-NO-releasing chitosan controls, with no associated cytotoxicity.

### 5.5. Chitosan-Graft Dendrimers

To further improve the NO payload, while simultaneously reducing the cytotoxicity of higher generations dendrimers, NO-loaded chitosan-graft-poly(amidoamine) (PAMAM) dendrimers (CS-PAMAM/NONOate) were prepared using a copper-catalyzed azide-alkyne cyclization reaction of azide-modified chitosan with a third generation alkynyl-PAMAM-G [21,119]. Compared to NO-releasing COS, which can release 0.16–0.52 µmol NO per mg COS/NO over 4 h, CS-PAMAM/NONOate can release ~1.5 µmol NO per mg of CS-PAMAM/NONOate within 2.5 h under similar conditions [21,113]. The antibiotic methicillin can be additionally loaded onto CS-PAMAM/NONOate through an amide reaction to further improve bacteria killing [119]. In these studies, 1 mg/mL CS-PAMAM/NO reduced *S. aureus* and MRSA viability by >99% and ~60%, respectively. At 2.5 mg/mL, CS-PAMAM/NO reduced *S. aureus* viability ~1 log more compared to CS-PAMAM treatment. With methicillin addition, 1 mg/mL CS-PAMAM/NO further reduced MRSA viability with ~80% cell death [21,119]. In in vivo rat wound infection models, the CS-PAMAM-MET/NONOate group showed the fastest healing rate after 10 d of treatment, with no apparent cytotoxicity [119].

### 5.6. NO-Releasing Alginate Scaffolds and Hydrogels

In NO-releasing alginate scaffolds, *N*-diazeniumdiolate NO donor groups can be introduced into alginate scaffolds via secondary amines, which are formed through carbodiimide chemistry that is used to modify alginate carboxylic acid groups with small molecule alkyl amines [120]. NO-release varied depending on the amine precursor structure in a manner similar to small molecule NO donors, with longer chain length alkyl amines having faster NO release. Further, the molecular weight (MW) of alginate had significant effects on NO release kinetics even with the same amine precursor, with high MW alginate increasing NO release half-lives. This enables synthesis of an alginate scaffold with a tunable NO release profile by modifying the ratio of low and high MW alginate used [120]. Higher concentrations of alginate materials with the fastest and slowest NO-releasing kinetics were required to achieve bactericidal effects compared to alginates with moderate NO release kinetics. This is due to premature NO release in the former and insufficient NO release in the latter, leading to lower therapeutic NO dose at the target site. Consistent with other studies on the size dependence of macromolecular scaffolds [124,126], at similar NO-release kinetics, NO-releasing alginate oligosaccharides (5 kDa) were more bactericidal than their high molecular weight counterparts (300 kDa). Alginate hydrogels containing silver nanoparticles (Ag NPs) and the NO donor *S*-nitroso-MSA have also been evaluated recently [99] and were bactericidal at low concentrations of 2 µg/mL hydrogel treatment against *S. mutans*, *S. aureus*, and *E. coli*. While the combination hydrogel was not cytotoxic at 2 µg/mL, it was toxic against Vero cell lines at concentrations above 6 µg/mL compared to non-Ag NP-containing or non-NO-releasing alginate gels.

### 5.7. NO-Releasing Cyclodextrins

In the case of cyclodextrins (CD), mono-substituted *β*-CD derivatives may be obtained via reaction with tosyl chloride under basic conditions and further reaction of tosyl groups to yield secondary amine groups. The NO load can be further increased by converting the secondary hydroxyl groups of *β*-CD into bromo groups that can be displaced with primary amines to yield secondary amine-modified hepta-substituted *β*-CD derivatives for diazeniumdiolate functionalization [122]. The NO payload and release from cyclodextrins is likewise tunable by altering the type and amount of amine precursor and chemical modification of the exterior surface of the cyclodextrin [122]. However, NO loading is not strictly governed by available surface chemistry. For example, hepta-substituted *β*-CD-derivatives with seven times more secondary amine sites have only 2–4 times higher NO payload than monosubstituted CD-derivatives, due to steric hindrance and repulsive interactions near the amine group. The NO dose at the MBC was similar for both hepta-substituted and monosubstituted *β*-CD-derivatives for the same amine precursor; although, lower concentrations of hepta-substituted *β*-CD/NO were needed for bacteria killing simply due to its higher NO load. Further, primary amine-terminated CD-NONOates required lower NO doses for *P. aeruginosa* eradication compared to methyl-, hydroxyl-, and methoxyl-terminated CD-NONOates, once again due to the association of primary amines with the bacterial membrane. *β*-CD scaffolds have an outer diameter of <2 nm, which can allow for better biofilm penetration and eradication regardless of the mechanical strength of the biofilm matrix. Indeed, while different concentrations of tobramycin and colistin were required to eliminate *P. aeruginosa* biofilms formed in different growth media, the same concentration of 2 mg/mL *β*-CD/NO can eradicate *P. aeruginosa* biofilms irrespective of growth conditions, suggesting that *β*-CD/NO and NO-based therapeutics are effective regardless of the physiological status of the bacteria within the biofilm [121]. However, it should be noted that *β*-CD/NO with a higher initial burst of NO had a higher cytotoxicity to L929 mouse fibroblast cells. To optimize between antimicrobial activity and safety, a balance between NO release, anti-microbial activity and cytotoxicity can be obtained through manipulating chemical groups and precursors associated with the cyclodextrin.

## 6. NO Delivery via Inorganic and Polymeric Nanoparticles and Nanocarriers

Different nanomaterials and nanoparticles (NPs) have been designed for NO delivery, including inorganic silica NPs, surface functionalized metal oxide NPs, dendrimers, and core cross-linked star polymers. The physicochemical properties of such NO-releasing NPs (NO-NPs), such as size, shape, charge, and hydrophobicity, may be tuned by varying their core material, synthetic precursors and processes, and through various surface functionalization [105,124,125,126,129,130,133,134,135,136]. These modifications can improve the specificity and interaction of NO-NPs with bacteria or biofilms, enabling targeted NO release near the site of infection and reducing cytotoxicity against mammalian cells.

### 6.1. NO-Releasing Silica Nanoparticles

NO-releasing silica NPs can be synthesized using sol–gel processes with diazeniumdiolate groups introduced to amine moieties through reaction with high pressure NO gas [31,104,123,124,125,126,127,128,157]. Initial studies found silica NO-NPs to be more effective against bacteria and less cytotoxic in vitro compared to small molecule NONOates, as only 70 µg/mL of AHAP3 NO-NP was required for 3-log killing of *P. aeruginosa* within 1 h in PBS while 2.5 mg/mL of Proli/NONOate were needed for the same effect. Further, AHAP3 NO-NP was not cytotoxic at concentrations up to 800 µg/mL while Proli/NONOate was cytotoxic at bactericidal concentrations [104].

#### Physical and Surface Properties of NP Affect Their Association with Bacteria and the Activity of Their NO-Releasing NO-NP Counterparts

The size, shape, and surface properties of the NO-NP have considerable effects on their activity and cytotoxicity [123,124,125,157]. MAP3 NO-NP was 1000 times more effective against *P. aeruginosa* biofilms than AHAP NO-NP at equivalent concentrations of 8 mg/mL [123]. This increase in killing could not be accounted for by the twofold increase in total NO released by MAP3 NO-NP alone, and it was hypothesized that the smaller size of MAP3 and possible difference in surface charge may influence biofilm penetration and bacteria association. Indeed, greater amounts of biofilm killing were correlated to higher levels of associated NPs, with the Gram-negative *E. coli* biofilms being more susceptible than biofilms of Gram-positive bacteria, such as *S. epidermidis* and *S. aureus*. At similar NO load and flux, AHAP NO-NPs of smaller sizes (50 nm) also had a lower minimal bactericidal concentration (MBC) of 0.8 mg/mL than AHAP NO-NPs of larger sizes (100 and 200 nm, MBC = 1.5 mg/mL) against *P. aeruginosa*. Similarly, silica NO-NPs of smaller sizes of 14 nm or a higher aspect ratio of 8 had lower MBC against planktonic and biofilm *P. aeruginosa* and *S. aureus*, although 14 nm and 150 nm NPs were more cytotoxic than 50 nm NPs [126]. Increasing the alkyl chain length in quaternary ammonium functionalized silica NO-NP reduced the MBC towards *S. aureus* but had no effect on the MBC towards *P. aeruginosa*, but did result in increased cytotoxicity [124]. Backlund et al. designed NO-NPs of similar sizes (~150 nm) with different aminosilanes that had different surface charges and NO-release kinetics [127]. AHAP3 NPs had a greater positive surface charge than MAP3 and AEAP3 NPs. AHAP3 NO-NPs had similar NO-release kinetics to MAP3 NO-NPs, but AEAP3 NO-NP showed a significantly longer NO-release half-life. Consequently, AHAP3 NO-NPs were more effective than MAP3 NO-NPs against periodontal pathogens due to higher bacterial association while AEAP3 NO-NPs were more effective than AHAP3 NO-NPs due to more sustained NO release. Similarly, silane-based hydrogel-glass composites with sustained NO release in the nM range over 24 h was effective against *C. candida*, *S. aureus*, and MRSA in vitro and in vivo mouse skin infection and rat central venous catheter biofilm models, as well as improving wound healing in the skin infection model [31,128,157].

### 6.2. NO-Releasing Polymeric Nanoparticles

Many studies have been carried out in recent years on NO-releasing polymeric nanomaterials and their use in the treatment of bacterial infections. These polymeric materials have been described in depth in a recent review by Rong et al. [158] and will be briefly touched on. More recently, NO-releasing polymeric scaffolds that incorporate other ligands such as antibodies, photoactivated modules, or surface charge switchable components have also been developed for antimicrobial purposes and will be described in more details.

#### 6.2.1. POEGMA Containing NO-Releasing NPs

The Boyer group used reversible addition−fragmentation chain-transfer (RAFT) polymerization to generate block copolymers on which other groups or compounds, such as antibiotics, may be conjugated, or to provide amine sites for the formation of diazeniumdiolate NO-donor groups [23,54,129]. Visible light-mediated aqueous RAFT polymerization-induced self-assembly (PISA) may also be utilized to perform the polymerization under mild room temperature conditions [129]. In these NO-releasing polymeric NPs, poly (oligoethylene glycol methacrylate) (POEGMA) is used to increase hydrophilicity when incorporated with hydrophobic blocks such as poly (vinylbenzaldehyde) (PVBA) or poly(glycidyl methacrylate) (PGMA).

Initial studies with P(OEGMA-b-P(VMD) core cross-linked star polymers with spermine and NO donor conjugation indicate a rapid initial burst of NO release, and sustained NO release over 70 h. The star polymer reduced *P. aeruginosa* biofilm formation in a concentration dependent manner with corresponding increases in planktonic cells up to 400 µg/mL, with planktonic cell viability reduced by 20% compared to untreated controls at 400 µg/mL [23]. Synergistic effects were observed when gentamicin was conjugated to a NO-releasing P(OEGMA)-b-PVBA block copolymer. Treatment with 10 µM of the compound resulted in a reduction in *P. aeruginosa* planktonic culture and biofilm viability by >90% compared to a 5–7% decrease in viability with 10 µM gentamicin treatment alone [54]. NO-release kinetics and *P. aeruginosa* biofilm dispersal activity of NO-releasing POEGMA-b-PGMA NPs were dependent on the shape of the NP, with ~600 µM of cumulative NO released within 1 h by spherical NPs (S-NO) and 5 h by worm-like NPs (W-NO) respectively. Consequently, W-NO was ineffective at dispersing *P. aeruginosa* biofilms (<10% dispersal) due to its slow rate of NO release while S-NO induced >70% reduction in biofilm within 30 min. However, when treatment time was increased to 60 min, W-NO dispersed 86% of the biofilm [129].

More recently, Adnan et al. grafted P(OEGMA)-b-P(ABA) onto poly dopamine (PDA) coated iron oxide NPs (IONPs) to generate a multifunctional NO-releasing nanoparticles suited for both combinatorial therapy and theranostic application via NO activity and magnetic field-responsive IONP [105]. Within 48 h, IONP@PDA-NO released larger amounts of cumulative NO (600 µM) at a more gradual rate than the P(OEGMA)-b-P(ABA) polymer-stabilized counterpart IONP@PDA-HP-NO (~100 µM NO by 48 h), which has a rapid rate of NO release. While both were able to induce *P. aeruginosa* biofilm dispersal at high NO concentrations (1.5 µM), only IONP@PDA-HP-NO could do so at lower NO concentrations (0.375 µM). It is hypothesized that the more stable IONP@PDA-HP-NO is smaller in size than IONP@PDA-NO, which tends to form aggregates, and penetrates the biofilm matrix more effectively for NO delivery.

#### 6.2.2. PGLA-Based NO-Releasing Nanoparticles

Poly(lactic-co-glycolic acid) (PGLA) is biodegradable and biocompatible. Its two degradation products, lactic acid and glycolic acid, are naturally processed and result in minimal systemic toxicities. As such, PLGA-based nano and microparticles were utilized and synthesized via emulsion solvent evaporation methods for the encapsulation and delivery of NO precursor ISMN or the NO donor polyethylenimine diazeniumdiolate (PEI/NONOate) [130,131]. ISMN encapsulated PGLA-based NPs displayed lower ISMN loading (0.4% vs. 2.2%, respectively) and slower initial NO release than corresponding microparticles (MPs) [130]. The ISMN encapsulated PGLA-based MPs had sustained NO release over 5 d and displayed in vitro activity against *S. aureus* at 7.5 mg/mL against planktonic cells and 30 mg/mL against biofilm cells. Higher concentrations were required than free ISMN, due to incomplete drug release from the MP. For PEI/NONOate doped PGLA NPs (PLGA-PEI/NO NPs), NO-release was evaluated in simulated wound fluid. Fast NO release (~85% of total loaded NO of 122 µmol/g) was observed over the first 24 h, with NO release was sustained over the subsequent 4 days. PLGA-PEI/NO NPs can effectively bind to the surface of MRSA biofilm matrix due to its cationic property (Zeta potential + 34.6) and displayed good activity in in vivo MRSA wound infections carried out using STZ-induced diabetic mice. In the model, by day 12 post infection, PLGA-PEI/NO NPs treated mice displayed a very small wound area and a lack of infection, suggesting that the MRSA biofilm was completely eradicated [131].

#### 6.2.3. Antibiotic Conjugated or Surface Charge Switchable NO-NPs with Bacteria and Biofilm Targeting Properties

Recently designed NO-NPs can more specifically target bacteria and biofilm through the conjugation of antibodies or through charge switchable components. Zhang et al. prepared ISMN loaded immunoliposomes by conjugating the anti-*S. aureus* α-toxin monoclonal antibody with liposomes that were previously reacted with glutaraldehye [136]. It is thought that compared with topical antimicrobials, immunoliposomes could better penetrate and be retained in the biofilm, which could then facilitate drug release in proximity of the bacteria over an extended duration. At 45 mg/mL, the ISMN loaded immunoliposomes (ISMN-lipo-AntiHLA) were more effective at preventing *S. aureus* biofilm formation (~2% biofilm remaining) compared to ISMN-liposomes or ISMN treatment (~18 and 36% biofilm remaining, respectively). Similarly, 45 mg/mL of ISMN-lipo-AntiHLA reduced the viability of pre-formed *S. aureus* biofilms by ~97%, while ISMN-liposomes or ISMN treatment had lower activity with ~77% or 63% inhibition, respectively. At lower concentrations of 11 mg/mL, all three treatments displayed similar effects on biofilm formation and *S. aureus viability*. The study highlighted immunoliposomes as a novel drug and NO delivery system for biofilm eradication; although, in vivo studies have yet to be carried out to assess their safety and efficacy.

**Figure 2:**
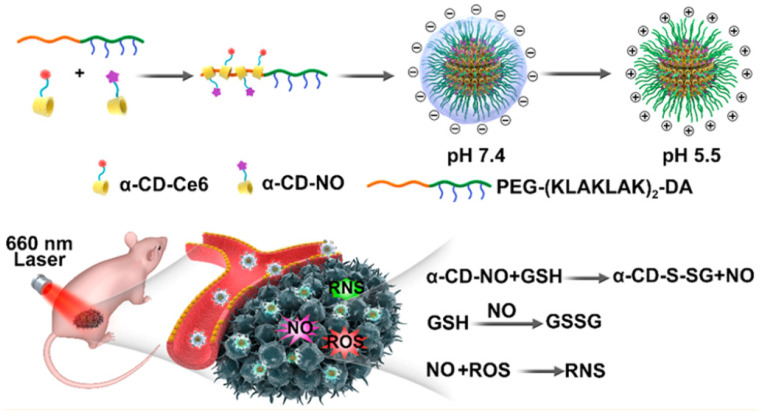
Synthesis, design, and mechanism of the surface charge switchable α-CD-Ce6-NO-DA, which carried a negative surface charge at pH 7.4, but positive surface charge at pH 5.5, in turn promoting efficient penetration of the supramolecular nanocarrier into biofilm. NO is released from α-CD-Ce6-NO-DA upon reaction with biofilm GSH, which depletes biofilm GSH. ROS is generated upon 660 nm laser irradiation, which could react with NO and further improve PDT efficiency. Reproduced with permission from [134].

Instead of incorporating positively charged groups or using NPs with positive surface charges, Hu et al. [134] and Liu et al. [133] designed charge switchable NO-NPs, α-CD-Ce6-NO-DA, and PGLA-PLH-PEG tri-block copolymer (PNBNPs), which have negative surface charges at pH 7.4 but become positively charged at acidic biofilm infection sites. The former (Figure 2) was fabricated via host–guest interactions between an α-cyclodextrin conjugated NO (α-CD-NO) or chlorin e6 (α-CD-Ce6) prodrug and the pH sensitive block polypeptide copolymer PEG(KLAKLAK)_2_-DA. Surface charge switching occurs due to the acid-triggered cleavage of the amide bond formed between an amino group and DA. Hu et al. has also made use of the overexpression of glutathione (GSH) within biofilms for targeted NO-release. NO release from α-CD-Ce6-NO-DA NO-NP is induced upon high GSH levels in biofilm, and the NO carrier is comparatively stable during bloodstream circulation or in healthy tissues, hence preventing off-target side effects of NO-release. In vitro, most NO was released from 2 mL of the micellar solution (100 µg/mL NO equivalent) in the presence of 8 µM GSH within 2 h, while only 3.2% of NO was release in the presence of 2 µM GSH in the same time span. This method has an advantage of depleting biofilm GSH levels, in turn improving Ce6-mediate photodynamic therapy (PDT) efficiency, as there is less GSH available to protect the biofilm against ROS generated with PDT. NO released can also react with ROS to generate RNS and further improve PDT efficiency. Furthermore, in a previous study by Friedman et al., it was reported that GSNO, which is formed following reaction of NO and GSH, potentiates and improve the antimicrobial activity of NO-NPs [159]. In combination, α-CD-Ce6-NO-DA can be effective at lower photosensitizer doses and laser intensity, hence reducing damage to healthy tissues. Indeed, at similar laser irradiation doses in vitro and in an in vivo mouse subcutaneous infection model, α-CD-Ce6-NO-DA displayed a much stronger bactericidal ability, lower cytotoxicity, increased wound healing, and reduced side effect to healthy tissue, compared to non-NO releasing NP controls and non-surface charge switchable designs. Other NPs with light triggered NO-release designs will be further discussed in the next section.

The PLGA-PLH-PEG tri-block charge switchable copolymer-modified *N*-diazeniumdiolate NP (PNBNPs) by Liu et al. consists of a *N*-diazeniumdiolate NO donor inner layer and a bacterial targeting PLGA-PLH-PEG polymeric outer shell [133]. PEG within the copolymer helps reduce nonspecific interactions of PLGA with tissue cells or bio-adhesion of PLGA containing NP in vivo and prolongs transmission circulation. Under acidic conditions, imidazole groups present on PLH accept protons, increasing the surface charge zeta potential from −7.5 mV at pH 7.4 to +20 mV at pH 5.0. At pH 7.4, NO release from the PNBNPs were fairly low with 1 µmol/mg of total NO released in 24 h and a half-life of ~4 h. In comparison, at pH 6, 3 µmol/mg total NO is released in 24 h with a NO-release half-life of 30 min. Bare, hollow NO-releasing P(AmEMA-co-EGDMA) NP, which do not have the charge switchable copolymer cladded onto its outer surface, had a NO release half-life of 20 and 10 min at pH 7.4 and 6, respectively. PNBNPs had no antibacterial effect at 3 mg/mL at pH 7.4. However, it was inhibitory towards *S. aureus* at 1.3 mg/mL (corresponding to 4 µmol/mL NO loading amount) at pH 6. It displayed no cytotoxicity towards L929 mouse fibroblasts when incubated at pH 7.4. In vivo mouse implant biofilm models, PNBNP-treated groups decreased *S. aureus* viability by 1.8 log compared to the saline control, 0.3 log compared to free vancomycin and 2 log compared to NO-releasing P(AmEMA-co-EGDMA) NP.

#### 6.2.4. NO-Releasing Materials and Photodynamic and Photothermal Therapy for Antimicrobial Treatment

Phototherapy methods, such as photodynamic therapy (PDT) and photothermal therapy (PTT) have been previously evaluated for use in antimicrobial and anti-biofilm treatments [64,65,135,160]. PDT and PTT can induce cell death. The former does so through the generation of ROS by a photosensitizer and the latter, through the generation of heat following light absorption. Although phototherapy offers non-invasive and precise spatiotemporal control over the treatment site, the high concentrations of photosensitizer or high temperatures of PTT required to eradicate mature biofilms may still lead to cytotoxicity and damage surrounding tissues. In addition, even at high concentrations, PDT or PTT alone may not be effective at eradicating biofilms [161,162]. Recent NO-delivery designs for biofilm eradication have, hence, incorporated either a photosensitizer or photocleavable modules. These designs make use of NO release to sensitize bacteria cells to ROS and heat stress, and in turn potentiate the effects of PDT and PTT. This enables biofilm eradication at lower PS concentrations, irradiation dose, and PTT temperatures, with targeted NO delivery upon irradiation and, in turn, reduced side effects. These NO-releasing systems were more synergistic and effective at eradicating *S. aureus*, MRSA, and *P. aeruginosa* biofilms both in vitro and in vivo, and had less cytotoxicity than relevant control groups, i.e., NO-delivery designs without photosensitizer or NO-releasing groups [22,133,134,135,136].

**Figure 3:**
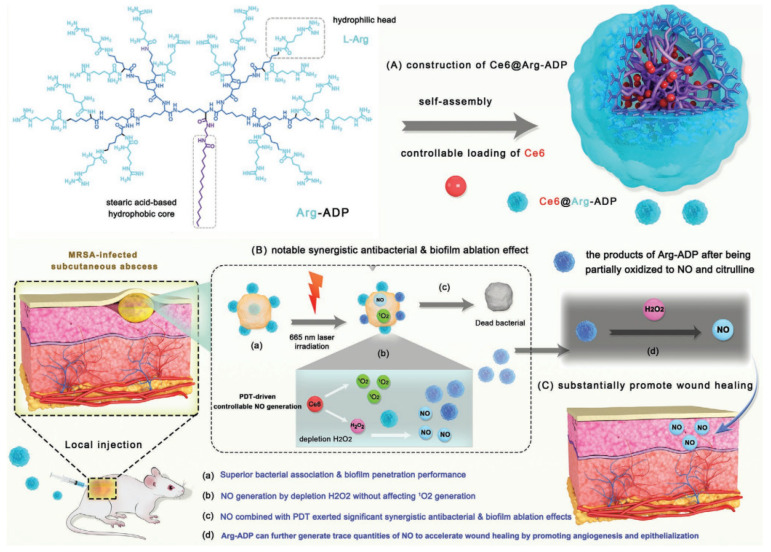
Preparation and mechanism of action of Ce6@Arg-ADP. NO release is tunable with NIR irradiation duration. The NO generated can both eradicate wound bacteria and promote wound healing. Reproduced with permission from [137].

Hu et al. made use of chlorin e6 (Ce6) as a high ROS-generating photosensitizer in α-CD-Ce6-NO-DA, for which the mechanism of biofilm eradication has been previously described [134]. Likewise, Zhu et al. designed a PDT-driven NO controllable generation system (Ce6@Arg-ADP, Figure 3) with L-Arg-rich amphiphilic dendritic peptide (Arg-ADP) as a carrier [137]. Enhanced antibacterial activity and biofilm penetration is achieved to a lesser extent via amino groups and to a larger extent through the presence of abundant surface guanidine groups of the Arg-ADP peptide that can associate with and promote bacterial cell rupture. Irradiation of Ce6@Arg-ADP resulted in the generation of H_2_O_2_ by PDT, which can oxidize Arg-ADP to NO and L-citrulline. The system is tunable as the amount of H_2_O_2_ and, in turn, the NO generated increases with increased Ce6 loading and near infrared (NIR) irradiation duration. In vitro, 1 mg/mL of Ce6@Arg-ADP + 665 nm laser treatment at an intensity of 115 mW/cm^2^ eradicated 90% of MRSA biofilms while in the in vivo subcutaneous abscess model, the same treatment eliminated all bacteria at the wound site. Further, following bacteria eradication, Arg-ADP can generate trace amounts of NO to facilitate wound healing through NO-induced angiogenesis and epithelialization of the wound tissues.

**Figure 4:**
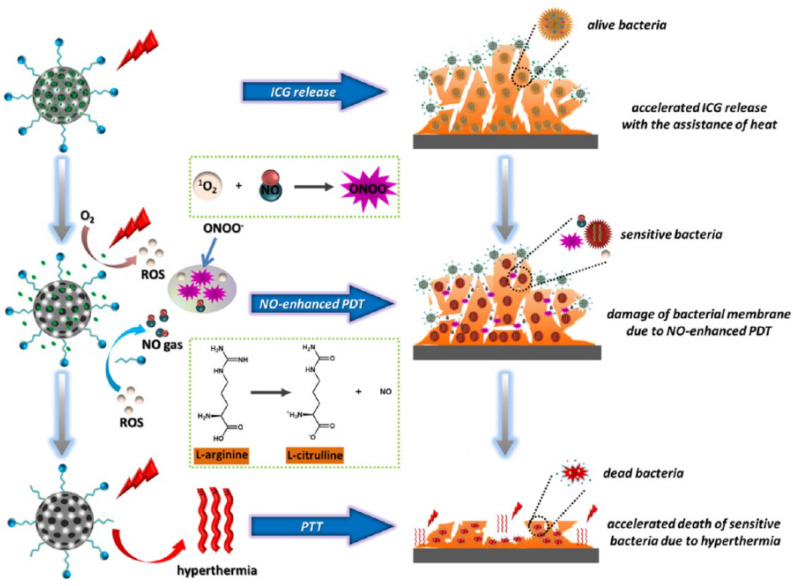
Mechanism of action of PDT-, PTT-, and NO-mediated killing of bacteria and eradication of biofilm by AI-MDPA. NIR irradiation generates heat, ROS, and NO that sensitizes bacterial cells to hyperthermia. Reproduced with permission from [135].

Yuan et al. combined PDT with low-temperature PTT by incorporating the photosensitizer indocyanine green (ICG) with the photothermal conversion efficient mesoporous polydopamine (MPDA) in an all-in-one phototherapeutic nanoplatform (AI-MDPA) (Figure 4) [135]. In a similar manner, Near Infrared (NIR) irradiation of AI-MDPA at 808 nm generates heat, inducing ICG release and ROS production, which in turn catalyzes NO release from L-Arg. NO production of NIR-triggered AI-MPDA is power-density and concentration dependent, and a higher laser power was used here compared to other studies [134,137]. A total of 4 µM NO was produced when 0.2 mg AI-MPDA was irradiated with NIR at 1 W/cm^2^. Laser treatment combined with 0.2 mg/mL AI-MPDA decreased in vitro *S. aureus* biofilms by 80% compared to control groups and enhanced bacterial cell membrane disruption and lysis. In an in vivo skin infection model, AI-MPDA + 10 min of 1 W/cm^2^ laser efficiently killed *S. aureus*, with accelerated healing, decreased wound area, and no associated cytotoxicity or pathological abnormalities. While a higher power density of 2 W/cm^2^ could eradicate all bacteria from the wound site, the mouse skin surface temperature increased from 45 to 50 °C compared to when 1 W/cm^2^ power density was used, and an obvious inflammatory response and toxicity was observed.

Sun et al. designed hierarchically structured nanoparticles (UCNP@PCN), composed of upconversion nanoparticles (UCNPs) and Zr(IV)-based porphyrinic metal–organic frameworks (PCN-224) that were doped with L-arg and incorporated into polyvinylidene fluoride (PVDF) matrix to obtain an electrospun nanocomposite membrane (UCNP@PCN@LA-PVDF). The electrospun nanocomposite membrane released ~0.37 µM of NO within 30 min of NIR irradiation at 980 nm and displayed strong bactericidal activity (>90% reduction in viability) against *P. aeruginosa* and *S. aureus*. No cytotoxicity against a L929 fibroblast cell line was observed. In vivo, UCNP@PCN@LA-PVDF promoted bacteria eradication and wound healing more than controls. NO can be release on demand by switching on/off the NIR light, enabling controlled NO delivery [136].

Unlike designs that incorporate NO donor groups via post-modification procedures, Shen et al. fabricated a poly(ethylene oxide)-b-PCouNO (PEO-b-PCouNO) amphiphilic diblock copolymer [22]. The nitrosamine-based NO donors can be polymerized into amphiphiles using RAFT polymerization and can self-assemble into micellar NPs in an aqueous solution. This can hopefully address the issue of incomplete post-modification with diazeniumdiolate or RSNO in other polymeric materials due to steric hindrance and the improved stability of NO-releasing moieties [123]. It also provides an option of easily incorporating antibiotics within the NP core, which can further enhance antibacterial and antibiofilm activities. In this design, CouNO act as an *N*-nitrosoamine-based NO donor containing a coumarin chromophore that is stable in the dark but exhibit visible-light-mediated NO release. When irradiated at 410 nm with a power density of 7.5 mW/cm^2^, 40 µM of NO was released in 40 min from 40 µM of the *N*-nitrosamine-based precursor. In micelle NP, ~110 µM of NO was released from 0.1 g/L of the NP, with increasing NO release from higher irradiation intensities. NO release from the micellar NP can efficiently disperse *P. aeruginosa* biofilm, with co-delivery of NO and ciprofloxacin enabling simultaneous biofilm dispersal and bacteria killing. Further, NO release was accompanied by strong increase in fluorescence, enabling simultaneous monitoring of NO release.

#### 6.2.5. NO-Releasing Dendrimers and Hyperbranched Polymers

Dendrimers are monodispersed, highly ordered, hyper-branched polymeric nanostructures with multivalent surfaces. Hyperbranched polymers have dendrimer-like properties but are polydispersed, and have irregular branching and structure, but are cheaper and easier to synthesize [163]. Both can store high concentrations of NO within a single molecular framework, and a NO content as high as 11.65 µmol/mg has been reported in the NO-releasing *β*-cyclodextrin-PAMAM star polymer [164]. Dendritic scaffolds containing multiple amine groups, such as polypropylenimine, amine-functionalized poly(propylene imine) (PPI) or PAMAM dendrimers, are especially suited the formation of NO-releasing diazeniumdiolates with NO payloads as high as 5.6 µmol/mg [103,138,164,165]. *N*-diazeniumdiolate-functionalized hyperbranched polyaminoglycosides can have a NO payload of 0.4–1.28 µmol/mg, depending on the amine content of the aminoglycoside monomer [41,166]. NO donors such as L-arg may also be incorporated into dendrimers [137].

In a study by Backlund et al., a PROLI/NO modified NO-releasing dendrimer (G1-PAMAM-PO/NO) had superior bactericidal activity against the periodontal pathogens *P. gingivalis*, *A. actinomycetemcomitans*, *S. mutans*, and *S. sanguinis* compared to MAP3 silica NPs, which released similar amounts of NO within a similar time range, and PROLI/NO controls, which release and store more NO than the dendrimer [139]. The difference in activity is likely because of the enhanced association of the dendritic scaffold to the bacteria [167]. NO-releasing hyperbranched polyaminoglycosides (h-polyaminoglycosides/NO) such as h-polykanamycin (h-PKA/NO) and PAMAM (h-PAMAM/NO) were also effective against periodontal pathogens [41,140,166].

Surface modification of dendrimers influences NO payload, NO release, dendrimer–bacteria association, antibacterial and antibiofilm activity, and cytotoxicity. In general, NO storage and NO flux increase as a function of dendrimer size (generation) and decrease in the presence of bulky or hydrophobic groups near the amine moiety [101,103,138,165,167]. However, the effects of those various modifications may not be significant. In fact, NO-releasing PAMAM dendrimers modified with different alkyl epoxides or groups with different hydrophobicities exhibited similar NO storage and payloads [100,101,138]. In h-PAMAM/NO, propylene oxide (PO) modifications decreased the NO release half-life as PO modifications replaced cationic amines that can stabilize the *N*-diazeniumdiolate cations [140]. This may affect activity and cytotoxicity, as described below. In h-PKA/NO, altering the terminal groups to ethylenediamine (EDA) or monoethanol amine (MEA) increased NO payload by four times compared to h-PKA/NO. While h-PKA/NO-EDA and h-PKA/NO-MEA display similar total NO payloads, NO release half-life of h-PKA/NO-EDA is more than two times longer than h-PKA/NO-MEA. Despite differences in NO load and release, h-PKA/NO, h-PKA/NO-EDA, and h-PKA/NO-MEA display similar MBCs activity against periodontal pathogens.

Amphiphilic and hydrophobic dendrimers, and dendrimers modified by the addition alkyl epoxides of longer alkyl chain lengths, have better anti-bacterial and antibiofilm activities due to their membrane disruption properties [100,101,138]. NO release may, or may not, improve bactericidal effects depending on the innate bactericidal activity of the dendrimer scaffold. For example, considering that NO payloads and kinetics are similar between QA alkyl chains functionalized PAMAM, NO release from short QA alkyl chains functionalized PAMAM improved bactericidal effects relative to the scaffold by 2–7-fold but provided negligible or no improvements in MBC and MBEC for long chain dodecyl QA G1 and G4 PAMAM dendrimer scaffolds, which were already highly bactericidal and have up to 800 times lower MBC than their corresponding unmodified G1 and G4 PAMAM scaffolds [102]. The same trend was observed for alkyl chain and exterior functionality modified PAMAM dendrimers, which have very different MBCs despite similar structures and NO release kinetics [101,138]. In the case of the L-Arg-rich amphiphilic dendritic peptides, described above, compared to the Ce6@Lys-ADP control, which is adsorbed onto MRSA cell surface, Ce6@Arg-ADP is internalized or tightly bound to MRSA, a factor that contributes to its high bactericidal activity upon irradiation and release of ROS and NO [137]. For h-polyaminoglycosides/NO, the high density of aminoglycosides, an antibiotic, can contribute to its antimicrobial activity [166]. Comparing activities of different dendrimers and dendrimer-like structures, it was observed that while 16 mg/mL of h-PKA/NO and h-PAMAM/NO displayed similar bacterial killing activity, the NO dose from h-PKA/NO was four times lower [41]. A lower concentration of h-PKA/NO (8 mg/mL) and corresponding lower NO dose (~1.92 µmol/mL) was also needed to kill *S. mutans* than G1-PAMAM-PO/NO (MBC = 48 mg/mL, NO dose = ~37 µmol/mL) under similar experimental conditions, indicating that h-polyaminoglycosides/NO may be advantageous over the other molecular designs [140,166].

Cytotoxicity associated with dendrimers could be mitigated by other chemical modifications, such as PEGylation, hydroxyl, and carboxylic acid termination and the addition of QA moieties, without significantly affecting the antibacterial activity of the dendrimer [101,168,169]. Besides having better bacterial association, amphiphilic PAMAM dendrimers with intermediate ratios of PO/ 1,2-epoxy-9-decene (ED) were also less cytotoxic to MEF cells compared to dendrimers containing a high ratio of hydrophobic ED groups [138]. NO release has varying effects, increasing G1 PAMAM cytotoxicity to HGF-1 cell lines for NO-releasing propyl dendrimers but reducing cytotoxicity in NO-releasing octyl- and dodecyl dendrimers [100]. In addition, while h-PKA-EDA and h-PKA-MEA were much less cytotoxic towards HGF-1 cell lines than h-PKA, the reverse is true for their NO-releasing counterparts, which were much more cytotoxic in in vitro experiments. h-PAMAM with PO modification (h-PAMAM-PO-1) were less cytotoxic over short durations due to the conversion of potent primary amine groups to secondary amines (11% conversion efficiencies) but display toxicity over extended treatment times. Additional modifications with *N*-diazeniumdiolate simultaneously render h-PAMAM-PO-1/NO less effective against MRSA as the negatively charged *N*-diazeniumdiolate diminished its physical association with bacteria and reduced amine-directed killing, while increasing cytotoxicity due to its high NO dose. Increasing the molar ratio of PO used led to consumption of secondary amines along the polymer backbone, and resulted in lower total NO in h-PAMAM-PO-2/NO compared to h-PAMAM-PO-1/NO. h-PAMAM-PO-2/NO has comparable MBC to h-PAMAM-PO-1/NO and G3-PAMAM/NO, but with minimal cytotoxicity. As such, h-PAMAM-PO-2/NO is advantageous over h-PAMAM-PO-1/NO and G3-PAMAM/NO for future utilization [140]. Increased cytotoxicity due to higher dendron generations can also be circumvented by conjugation of low generation dendrons to a core molecule to form a star-shaped copolymer or grafting of higher generation PAMAM to chitosan [21,164].

Overall, NO-releasing dendrimers have potential as anti-bacterial and antibiofilm agents. However, care must be taken to balance the trade-offs between different modifications on antibacterial activity, NO release, and long-term cytotoxicity for its successful application as a therapeutic. Newer peptide dendrimers that are PDT activated may offer further advantages including controlled- and targeted-NO release and synergistic effects with PDT-generated ROS [137] and may serve as a basis for future developments of NO-releasing dendrimers.

### 6.3. NO-Releasing Gel, Polymers, and Coatings

Passive anti-fouling surfaces and the active release of biocidal agents can be utilized to prevent bacterial adhesion and biofouling. The former can reduce adhesion but generally does not kill adhered microorganisms, while biocidal agents may be limited by their concentrations resulting in a finite duration of action. Combining active NO-releasing materials with a passive anti-fouling surface coating may, hence, offer a synergistic approach to prevent surface fouling. Additional surface coatings may also influence NO release kinetics and reduce NO leaching [141,143,145,146,147]. In cases of blood-contacting medical devices, beyond prevention of bacteria fouling, NO-releasing surfaces can be designed to generate a constant flux of NO at concentrations representative of endothelial NO release for positive effects on blood vessel homeostasis and inhibition of platelet activation that might otherwise lead to thrombus embolism and lethal cardiovascular effects.

NO-releasing surfaces or coatings can be obtained through several methods. NO donors such as SNAP can be immobilized within Carbosil 2080A or silicone polymers through solvent evaporation or polymer swelling processes [144,145,146,147,148]. In the former, 10 wt% SNAP is often incorporated into Carbosil 2080A [144,145,146,170]. The Carbosil 2080 polymer has good biocompatibility over other PVC-based polymers, and its low water uptake minimized SNAP leaching into solution [171,172]. Further, SNAP can form orthorhombic crystals at concentrations above 4 wt%, which exhibit increased shelf stability, slower dissolution, and enables long term NO release. In all cases, Carbosil-SNAP coated surfaces can significantly reduce bacterial attachment compared to untreated control surfaces. For example, Carbosil-SNAP (20 wt%) reduced *P. aeruginosa* and *P. mirabilis* on a catheter surface by 2 and 3 log units, respectively, over 14 d, while CarboSil SNAP (10 wt%) reduced *E. coli* and *S. aureus* adherence by >95% over 24 h in LB medium at 37 °C [144,146]. The application of anti-fouling topcoats can further reduce bacteria adherence as described below.

Secondly, RSNO groups can be yielded on surfaces following plasma treatment and polymerization of thiol monomers and nitrosation [151,173]. Plasma polymerization offers an easy tunable method to increase NO loading, increasing polymer film thickness [151]. The increase in NO loading correlates with improved anti-biofilm activity, as Sadrearhami et al. showed that at 24 h, 250 nm and 500 nm film thickness reduced *P. aeruginosa* biofilm formation by 55% and 81%, respectively. Similarly, at 6 h, 250 nm film thickness reduced biofilm more than 125 nm film thickness, and at 36 h, 1000 nm film thickness further reduced *P. aeruginosa* biofilm as compared to 500 nm film thickness [151].

NO-releasing polymer coatings can also be grafted on relevant implant materials. For example, Hou et al. [24] used a precision-structured coating, termed (H(N)-b-S), composed of a both a surface antifouling block of poly(sulfobetaine methacrylate) (poly(SBMA)) and a subsurface bactericidal block of RSNO-modified NO-emitting functionalized poly(hydroxyethyl methacrylate) (poly(HEMA)) that was covalently grafted from polyurethane (PU) to prevent leeching or solvation of the coating material. While other hydrophilic antifouling surfaces will be discussed in more detail below, the (H(N)-b-S) material, with its hydrophilic outer surface and NO-releasing properties, displayed excellent short- and long-term broad-range antibacterial efficacies in vitro and in vivo, and was active against pan-resistant *P. aeruginosa*, carbapenem-resistant *K. pneumoniae*, MDR *A. baumannii*, and MDR *E. coli*. The H(N)-b-S coating also retained about 99.9% inhibition against MRSA and *P. aeruginosa* over 30 days in vitro and had no MRSA biofilm over 5 d in vivo. For the latter, the mean arterial pressure pig implanted with the H(N)-b-S coated catheter during anesthesia was above the lower normal limit and may be an indication of transient systemic toxicity of NO combined with anesthesia. However, beyond that, the coating had good antithrombogenicity and biocompatibility and is scalable in production and remains a promising antibacterial and antifouling coating.

Coatings of *N*-diazeniumdiolate-modification of silane xerogels may be obtained via sol–gel processes. The xerogels display high material stability with no change in surface properties after high pressure NO treatment [174]. NO flux from xerogels is easily tunable via the use of different ratios of different silane monomers or application of a topcoat [141,150]. In general, a higher reduction in bacterial and fungal adhesion is observed with xerogels with higher NO flux [141,142,175].

Lastly, uniform adherent *N*-diazeniumdiolate-functionalized polydopamine (PDA) films can be obtained by self-polymerization of dopamine on various surfaces through a simple immersion process. PDA can be easily functionalized with nucleophiles via Schiff base or Michael addition reactions, enabling easy introduction of both *N*-diazeniumdiolate groups and topcoats such as polyethylene glycol (PEG) or polytetrafluoroethylene (PTFE) onto the PDA surface [146,149]. Repeated coatings of *N*-diazeniumdiolate-functionalized PDA have been shown to have higher NO flux and improved anti-biofilm activity, with 2S-NO (repeated coating steps two times) and 5S-NO (repeated coating steps five times) reducing adhesion of *P. aeruginosa* by 77 and 97% compared to unmodified glass controls by 6 h and by 35 and 78% by 24 h. As in other cases, inclusion of the hydrophilic PEG topcoat further reduced adhesion and viable cell counts [149].

As mentioned above, NO-releasing polymers may be further coated with anti-fouling surfaces. Such topcoats may be hydrophobic or hydrophilic and could have rough or smooth surfaces. The coatings may be applied via spray-coating [141], spin-coating [145], dip-coating [146], soaking or adsorption [147,170], or incorporated within a structured copolymer [24].

The surface of hydrophilic coatings tends to be smooth, allowing for the formation of a hydration layer that can repulse non-specific proteins to prevent fouling [176]. A slippery surface also reduces surface area for protein and bacteria adhesion. In line with this, Homeyer et al. [147] used a simple immersion method to infuse NO-releasing catheters with silicone oil, which provides a super slippery hydrophobic surface. SP60D60 polymer, PEG, and Poly(SMBA) incorporated within a diblock copolymer brush have been used as hydrophilic topcoats with no significant effects on NO release kinetics [24,145,149]. In all cases, NO-releasing polymers with an anti-fouling topcoat display better antibacterial and anti-biofilm activity than the NO-releasing surfaces alone.

Random roughness of hydrophobic surfaces may promote protein attachment through increased surface area, van der Waals forces and electrostatic force. However, superhydrophobic materials with high surface roughness and low surface energy can also resist bacterial or platelet adhesion through the formation of an air layer, known as a plastron, at the surface–water interface [141,146]. Hydrophobic topcoats such as fluorinated silane/silica composite mixture have nano- and micro-scale roughness, which enhances superhydrophobicity, while other hydrophobic materials such as PTFE can be immobilized without harsh chemical treatment via the addition of a PDA coating. Both can be applied easily by spray and dip coating. In general, the presence of a hydrophobic coating reduced the initial NO burst and enables a steadier rate of NO release throughout the duration of evaluation by hindering the diffusion of water to the NO donor [146,147,170,177]. The initial NO burst may result in cytotoxicity and limit later NO release, hence limiting this burst effect is desirable. The topcoat also reduces leaching of SNAP from the polymer [141,146,147]. As such, hydrophobic topcoats may be a useful strategy to extend the release kinetics of NO donors.

Besides inorganic or synthetic topcoats, Devin et al. have also made use of hydrophobin SC3, a self-assembling amphiphilic protein produced by filamentous fungi, to form an extremely stable hydrophobic or hydrophilic monolayer coating on hydrophilic and hydrophobic surfaces, respectively [170]. An SC3 coating offers an easily applied eco-friendly option for anti-fouling purposes, and viable adhered *S. aureus* was found to be 50% on NO-releasing coating with SC3, compared to the control coating without NO release. Together with NO release, the coating was also able to further reduce fibrinogen and platelet adherence. The coating was also not cytotoxic to mouse fibroblast cells.

## 7. Conclusions and Future Perspectives

In this review, we have summarized current research on low molecular weight NO donors and NO-releasing polymeric materials used for the eradication of bacteria or biofilms or for the prevention of surface fouling. Among which, development of NO-releasing agents that have photo-responsive or bacteria-targeting properties have improved the specificity of NO delivery and have enabled spatial or temporal control over NO release. Combinatorial treatment with NO and antibiotics has also enhanced bacteria killing. Studies are increasingly incorporating in vivo infection models or animal models to evaluate the efficacy of the NO-releasing material in an infection setting or to investigate the systemic effects of NO release. In addition, more studies have made use of MDR strains or clinical isolates that would be relevant for the application of these NO-releasing materials in a clinical setting. At present, there are still limited studies on the use of NO on mixed species infection or biofilms, which may be important given the differences in strain response and tolerance to NO. However, the use of NO remains promising given the success of inhaled gas in clinical trials for the treatment of lung infection, which are generally caused by a variety of bacterial and fungal species, in CF patients. Taken together, the most promising approach for future developments this area would be to improve the NO-mediated killing of bacteria though antibiotic codelivery, PDT, or PTT, and to improve specificity in NO delivery to reduce NO-mediated off-target side effects. Furthermore, in vivo studies should be carried out to demonstrate biosafety and efficacy of the drug in a mammalian system, and the stability and shelf-lives of the compounds should be considered.

The kinetics of NO release play an important role in its antibacterial and anti-biofilm activity, and NO levels must reach the appropriate therapeutic doses for the eradication of bacteria or biofilm. In this sense, NO donors that rapidly release NO in solution may not be useful given that NO may be depleted by the time the donor gets to its target site. The use of NO donors or polymeric materials that enable targeted NO release can circumvent this issue by releasing NO close to the infection site. Alternatively, NO-releasing polymeric materials with higher NO storage and payload may be used to increase NO delivered to the targeted site. On a similar note, while burst release of NO may be useful for bacterial eradication, a more sustained NO release is beneficial for promoting other processes, such as wound healing, or to prolong anti-fouling effects of NO-releasing surfaces. More studies could be carried out to compare between different NO-releasing materials or NO donors to determine the optimal treatment regime for each circumstance.

## Figures and Tables

**Table 1:** Summary of NO concentration and its general effects on antibacterial and anti-biofilm activity and biosafety.

	Targeted Delivery of NO to Infection Site	Spontaneous NO Release
High NO concentration	Depending on kinetics of NO release, killing of planktonic bacteria and eradication of biofilms can occur at sufficiently high NO concentrations Lower likelihood of side effects due to NO, as NO is delivered and consumed close to or at the target site Side effects/toxicity caused by the drug delivery vehicle needs to be further evaluated	Off target NO release may lead to NO levels below the required therapeutic dose at the target site Higher potential of side- or systemic effects due to NO as it is spontaneously released Side effects/toxicity caused by the drug delivery vehicle needs to be further evaluated
Low NO concentration	Depending on kinetics of NO release, targeted NO release may enable local NO concentrations to reach low NO concentrations required for biofilm dispersal Lower potential of side effects due to NO as low dose of NO delivered, with NO delivery close to or at the target site Side effects/toxicity caused by the drug delivery vehicle needs to be further evaluated	

**Table 2:** Activity of low molecular weight NO donors on planktonic and biofilm bacteria.

NO Donor	Concentration of NO Donor/NO	Stains/Test Conditions	Activity	Ref.
Gaseous NO	Continuous 200 ppm NO or intermittent 300 ppm NO	In vitro *P. aeruginosa*, *S. aureus*, clinical strains In vivo *K. pneumoniae*, MDR *Klebsiella* and *S. aureus*	Killing of *P. aeruginosa*, *S. aureus*, *E. coli*, *K. pneumoniae*, and *B. cepacia* Improvement in lung function in murine lung infection models Reduce wound bacterial content in wound infection models	[56,57]
	Intermittent 160–240 ppm NO	Clinical trials and case studies *M. abscessus, E. coli, P. aeruginosa*, antibiotic-resistant *P. aeruginosa*, antibiotic-resistant *B. multivoran*, *S. aureus*, and Group B Streptococcus	Low dose (10 ppm)—Dispersal of biofilm aggregates in CF lungs High intermittent dose (160 ppm)—Eradication of bacteria and fungi and improved lung function	[32,58,59,60,61,62]
Metal nitrosyl complexes (e.g., Sodium nitroprusside (SNP))	25 µM–100 mM SNP (≈25 nM to 100 µM NO); 5–80 µM SNP (12 h treatment time)	In vitro *P. aeruginosa*, *S. marcescens*, *V. cholerae*, *E. coli*, *F. nucleatum*, *B. licheniformis*, *S. epidermidis*, *C. albicans*, and isolates from MBR and RO membrane	Inhibition of biofilm formation Induction of biofilm dispersal (25 to 500 nM SNP) Promotion of biofilm formation at higher concentrations (>25 mM SNP) Potentiate antimicrobial treatment (500 nM SNP)	[6,42,63]
Photoresponsive ruthenium compounds	µM	In vitro *S. epidermidis*, *P. aeruginosa*	Bactericidal activity (0.1 µM to > 1 µM) Codelivery with methicillin increased *S. epidermidis* bacteria/biofilm sensitivity to treatment (0.1 µM) *P. aeruginosa* biofilm eradication (20 µM) Selective killing of bacteria can be achieved through positively charged groups present on compounds (20 µM)	[64,65]
*S*-nitrosothiols (RSNO) (e.g., GSNO, SNAC)	nM–mM	In vitro *P. aeruginosa*, *S. marcescens*, *V. cholerae*, and Keratitis-causing isolates	Inhibition of biofilm formation (nM–µM RSNO) Induction of biofilm dispersal (nM–µM RSNO) Bactericidal against bacteria (mM RSNO)	[6,63,66]
*N*-diazeniumdiolates (NONOates)	10 pM–80 µM (Varying treatment duration and dosing regimens)	In vitro Isolates from MBR and RO membrane, *S. enterica*, *E. coli* O157:H7, *P. aeruginosa*, and CF isolates	Dispersal of biofilm cells Reduction in live cells	[42,67,68]
Furoxans	5–500 µM	In vitro *P. aeruginosa*	Biofilm dispersal for fast-NO releasing furoxan Inhibition of bacteria growth and eradication of bacteria in an NO independent manner	[69,70]
Antimicrobial-NO donor hybrid donors	nM–µM	In vitro *P. aeruginosa, S. pneumoniae, Haemophilus influenzae* (NTHi), clinical CF isolates of *P. aeruginosa*, *S. aureus*, and *E. coli* In vivo *S. aureus*	Dispersal of biofilms Eradication of biofilm with or without additional antibiotics (Depending on generation and design for C3Ds) Increasing susceptibility of biofilms to antibiotic treatment Synergistic effects when used with antibiotics	[25,26,71,72,73,74,75]
QS inhibitor—NO hybrid donor	150 µM	In vitro *P. aeruginosa*	Biofilm inhibition Some designs bactericidal against planktonic bacteria	[76]

**Table 3:** Activity of various NO-releasing polymeric materials on planktonic and biofilm bacteria.

Macromolecular NO-Releasing Material	Concentration of NO/NO Donor Used	Stains/Test Conditions	Activity	Ref.
NO-releasing chitosan oligosaccharide (COS/NO)	0.12–3.1 µmol NO/mL	In vitro Mucoid/non-mucoid/clinical *P. aeruginosa*, *E. coli*, and *S. aureus*	Killing of planktonic and biofilm cells, with complete eradication of biofilms at high COS/NO concentration Reduction in bacteria adhesion Synergistic effects when used with antibiotics Non-cytotoxic to mouse fibroblast at bactericidal concentrations	[40,113,114,115]
NO-releasing chitosan gels	Variable depending on design and NO donors used. NO concentrations of ~ nmol NO/mg film or initial NO flux of ~ nmol cm^−2^ min^−1^	In vitro *S. aureus*, *P. aeruginosa*, MRSA, *L. monocytogenes* and *E. faecalis* In vivo MRSA	Reduction in bacteria viability and in biofilm cells Synergistic antimicrobial effects when used with antibiotics Improved wound healing in gels used in in vivo wound infection models	[13,116,117,118]
NO-releasing chitosan-dendrimer (CS-PAMAM/NO)	1–2.5 mg/mL chitosan dendrimer (~1.5 µmol NO/mg)	In vitro *E. coli*, *S. aureus* and MRSA In vivo MRSA	Killing of planktonic and biofilm bacteria cells, with increased killing compared to CS/PAMAM backbone Reduction in biofilm biomass Improve wound healing rate in in vivo models Not cytotoxic to NIH/3T3 cells at 1 mg/mL	[21,119]
NO-releasing alginate	~ µmol NO/mL for NONOate conjugated alginate	In vitro *P. aeruginosa, S.* aureus, *B. cepacia* complex, MRSA, *S. mutans*, and *E. coli*	Killing of planktonic and biofilm bacteria cells Lower MW NONOate conjugated alginate most effective with higher effectiveness of than tobramycin treatment AHG-SN-MSA-AgNPs not cytotoxic to Vero cells at concentrations for bacterial eradication, but cytotoxic at >20 μg/mL	[99,120]
NO-releasing cyclodextrins (NO/CD)	100–2000 µg/mL NO/CD (~ nmol NO/mL)	In vitro *P. aeruginosa*	Killing of planktonic and biofilm bacteria, with lower hepta-substituted CD concentration needed due to its higher NO burst More effective than tobramycin and colistin treatment Not cytotoxic against L929 cell lines at up to 2000 µg /mL	[121,122]
NO-releasing silica NPs (NO-NPs)	~ µg/mL to mg/mL NO-NPs with varying NO release kinetics and flux	In vitro *P. aeruginosa, E. coli, S. aureus, S. epidermidis*, *S. aureus, A. actinomycetemcomitans, P. gingivalis, and S. mutans*	Killing of planktonic and biofilm bacteria cells Combining NO release and QA-functionalities can increase bactericidal efficacy Smaller NO-NP size and higher aspect ratio give lower MBC Cytotoxicity towards L929 fibroblast and HGF-1 cells varies, and is influenced by concentration used, quaternary ammonium (QA) functionalization with increased cytotoxicity at longer alkyl QA chain length, and whether there is NO release	[104,123,124,125,126,127]
NO-releasing silane-based hydrogel nanoparticle platform	Steady state NO in nM range	In vitro MRSA and MSSA *S. aureus* In vivo MRSA	Inhibition of MRSA and MSSA strains from 312 to 2500 μg/mL Prevention of biofilm in vitro and in in vivo rat central venous catheter biofilm model Promotion of wound healing in wound infection models	[31,128]
NO-releasing P(OEGMA) containing polymeric nanoparticles	Variable, dependent on design (see activity for more details)	In vitro *P. aeruginosa*	**Gentamicin-NONOate NPs block copolymer NP** Synergistic when co-delivered with antibiotics with gentamicin Induction of biofilm dispersal at lower concentration (5 mM) and biofilm cell death at higher concentrations (10–50 mM)	[54]
			**Spherical (S-NO) and worm-like NO-NPs (W-NO)** NO release dependent on morphology Biofilm dispersal and eradication	[129]
			**NO releasing polydopamine (PDA)-coated iron oxide NPs** Biofilm reduction at low NO concentration (0.375 µM NO)	[105]
			**Core cross-Linked star polymers** Biofilm reduction with 57–400 μg/mL NO star polymer (NO release of 886 nM/h/mg/mL; Rapid release within the first hour and sustained release over 70 h)	[23]
NO-releasing polymeric nanoparticles, microparticles, and liposomes	NPs and MPs used in mg/mL range	In vitro *S. aureus*, MRSA	Planktonic and biofilm cell eradication Biofilm dispersal	[130,131]
			Targeted delivery of NO possible via conjugation of antibodies or with charge switchable designs	[132,133]
Photo-activated NO-releasing polymeric materials	Variable, dependent on design (see activity for more details)	In vitro *P. aeruginosa, S. aureus*, MRSA, *E. coli* In vivo *S. aureus*, MRSA	**Self-assembled micellar NPs with hydrophobic antibiotic in core** ~110 μM NO released from 0.1 g/L micellar NP NO release dependent on irradiation time and intensities Biofilm reduction concentration and irradiation dependent Synergistic effect with antibiotic treatment Some cytotoxicity to HeLa and NHLF when used at 0.4 g/L	[22]
			**Surface charge switchable, GSH activated α-CD-Ce6-NO-DA** NO release dependent on GSH levels α-CD-Ce6-NO-DA (~10 μg/mL Ce6 and ~20 μg/mL NO) bactericidal with low laser irradiation More rapid wound healing with α-CD-Ce6-NO-DA	[134]
			**Phototherapeutic nanoplatform AI-MPDA** AI-MPDA + NIR irradiation (45 °C) (4.0 μM NO, 0.2 mg/mL AI-MPDA) bactericidal and decreased biofilm Enhanced bacteria killing and wound healing in vivo Limited cytotoxicity against NIH-3T3 fibroblasts with 0.05–0.5 mg/mL AI-MPDA with no toxicity observed in vivo	[135]
			**Electrospun nanocomposite membrane (UCNP@PCN@LA-PVDF)** Enhanced killing of bacteria with PDT and NO treatment Decreased bacteria and complete wound healing by day 7 in vivo No cytotoxicity against L929 fibroblasts and in vivo	[136]
			**PDT-driven NO controllable generation system (Ce6@Arg-ADP)** Enhanced killing of planktonic bacteria (8 µg/mL Ce6@Arg-ADP + laser) MRSA biofilm eradication with 1 mg/mL Ce6@Arg-ADP + laser Eradication of all bacteria in subcutaneous abscess with 1 mg/mL Ce6@Arg-ADP + laser treatment in vivo with no biotoxicity	[137]
NO-releasing dendrimers	Variable, dependent on design. ~0.69 to 1 µmol NO/mg dendrimer released over 2–4 h in PBS, pH 7.4, 37 °C with max. flux of 2400–15,000 ppb/mg	In vitro *P. aeruginosa*, *S. mutans*, *S. aureus*, *S. sanguinis*, *A. acetinomycetemcomitans*, and *P. gingivali*	Reduction in planktonic and biofilm cell viability at µg/mL dendrimer corresponding to nmol/mL NO dendrimer) NO releasing dendrimers may be more or less cytotoxic than dendrimer scaffolds depending on design	[100,102,138,139]
NO-releasing hyperbranched dendrimers	NO storage and NO release ~µmol/mg with half-life ranging from 28 to 80 min depending on design and modifications	In vitro *P. gingivalis*, *A. acetinomycetemcomitans*, *S. mutansm*, *S. viscosus*, and ex vivo multispecies subgingival biofilms	Eradication of planktonic and biofilm cells Reduction in biofilm metabolic activity Antimicrobial activity dependent on aeration condition, with less activity under anaerobic conditions	[41,140]
NO-releasing xerogels and polymer coatings	Variable, dependent on pH, coating, and media (see activity for more details)	In vitro *P. aeruginosa*	**Super-hydrophobic NO-releasing xerogels with fluorinated silane/silica composite topcoat** NO flux 60–53 pmol/cm^2^/s (6–24 layers of coating), with NO release duration extended from 59 h (no coating) to 105 h (12 layers) Reduction in bacteria surface adhesion and biofilm formation	[141]
			**NO-releasing (poly)acrylonitrile (PAN/NO) polymer** In PBS, initial NO burst of 3.2 nmols/min/mg; 24 h steady state NO ~ 17 pmol/min/mg and cumulative NO over 6 h at 25 nmol/mL Reduction in bacteria CFU with 3–10% w/v PAN/NO in PBS, but limited activity in TSB (7.5 nmol/mL cumulative NO over 6 h) Reduction in biofilm formation with 0.1–3% PAN/NO in TSB over 24 h Dispersal of biofilm with 1–3% w/v PAN/NO in PBS Synergistic effects when used with antimicrobials	[142]
			**NO-releasing coatings on PET and silicone elastomer** Reduction in viable bacteria	[143]
SNAP-containing Carbosil 2080A polymer (Carbosil-SNAP) with different top coats		In vitro *P. aeruginosa*, *P. mirabilis*, *S. aureus*, *E. coli*	Reduction in bacteria surface adhesion with following designs: 20 wt% Carbosil-SNAP with hydrophobic CarboSil topcoat (NO release >0.5 nmol/cm^2^/min for 3 weeks (physiological conditions)) Hydrophilic SP60D60 polymer topcoat on Carbosil-SNAP Antifouling PTFE immobilized on PDA anchor layer atop 10 wt% Carbosil-SNAP (NO surface flux of 0.05 nmol/cm^2^/ min over 5 days) Reduction in platelet adhesion in 3	[144,145,146]
SNAP-impregnated silicone catheters	NO release ~0.04 nmol/cm^2^/mL over 60 days or ~ >0.07 nmol/min/cm^2^ over a month	In vitro *P. aeruginosa, P. mirabilis, S. aureus, S. epidermidis*	Reduced bacteria adhesion and biofilm formation over 24 h–14 days	[147,148]
Other NO-releasing surfaces	NO flux in µM range (PBS, pH 7.4, 37 °C)	In vitro *P. aeruginosa* *S. aureus*	**NO-releasing polydopamine (PDA) coating with PEG grafted onto PDA** Reduction in bacteria adhesion with more PDA coatings and PEG grafting further inhibiting biofilm formation	[149]
			**NO-releasing titanium surfaces** Reduced bacteria adherence to AHAP/NO and AUTES/NO surfaces No cytotoxicity observed against human primary osteoblasts	[150]
			**Thiol-functionalized coatings** Improved NO loading with higher film thickness with corresponding improvement in inhibition of bacterial attachment to the surface	[151]
	NO release sustained over 15 days at levels >1 nmol/cm^2^/min and a maximum flux of ~ 3 nmol/cm^2^/min within <15 min	In vitro *S. aureus, S. epidermis, E. faecalis*, *P. aeruginosa*, *K. pneumoniae*, *A. baumannii*, and *E. coli* and relevant MDR isolates, In vivo (Murine subcutaneous infection model) *P. aeruginosa*, *A. baumannii*; (Porcine central venous catheterization model) N/A	**Precision-structured diblock copolymer brush (H(N)-b-S)** Surface antifouling block (S) and subsurface NO-releasing bactericidal block (H(N)) H(N)-b-S coating effective in inhibition of Gram-positive and Gram-negative in vitro and in vivo No toxicities against multiple cell lines, with H(N)-b-S coatings additionally showing no thrombus formation, low lymphocyte activation, and low protein fouling in vitro and biocompatibility in vivo	[24]

**Table 4:** Comparison of advantages and disadvantages of each NO-donor and NO-delivering polymeric material design.

NO Donors/Polymeric Materials	Advantages	Disadvantages
NO gas	FDA approved; Direct NO delivery to lung infection sites and surface of wound infections; Side effects easily reversed by stopping NO gas	React with oxygen to give potent pulmonary irritants like NO_2_ and with hemoglobin to give methemoglobin
Metal-nitrosyl complexes	Metal-nitrosyl complexes, such as sodium nitroprusside (SNP), is FDA approved and long history of use clinically	Possibility of cyanide toxicity when using SNP for prolonged treatment
Ru-nitrosyl complexes	Photo-responsive	Relatively new and less well studied for antimicrobial purposes
S-nitrosothiols (RSNO)	Present endogenously; Some, such as GSNO, have well studied metabolism and low toxicity; NO release can be modulated through various means, including light irradiation; Easily incorporated into polymeric scaffold	Spontaneous release of NO and formation of disulfide bonds in solution; Trans-nitrosylation reaction with other thiol groups present in the body; Multiple mechanisms of degradation by bacteria
*N*-diazeniumdiolates (NONOates)	Broad range of reproducible NO release kinetics; Easily incorporated into polymeric materials containing amine moieties by passing NO gas at high pressure; Stable in powder form and in alkaline solutions	Spontaneous NO release in solution under physiological conditions. Not used clinically
Furoxans	Well-studied NO release with applications in various NO mediated biological processes; Prolong duration of action compared to other NO donors; Thermally stable; May be conjugated to other groups for codelivery of antimicrobials and NO donor	Appears to have other non-NO dependent effects on evaluated bacteria (i.e., *P. aeruginosa*) that is not well studied or explained with NO release
Hybrid NO donor	Targeted NO release using antibiotics or antimicrobial peptides; Synergistic effect at eradicating bacteria/biofilm with both targeted NO release and QS inhibition or antimicrobial action	Earlier generations of some hybrid NO donors, such as C3D, require induction of *β*-lactamase production for activity
*NO-releasing polymeric materials*		
Chitosan-based NO-releasing materials	Chitosan scaffold is biodegradable, biocompatible and has innate antimicrobial activity; Cationic chitosan promotes association with negatively charged bacterial membranes; Primary amine groups offer a straightforward means of incorporating NO-releasing moieties	In cases like NO-releasing chitosan oligosaccharide (COS/NO), cationic chitosan may improve cohesion of negatively charged biofilms
Alginate-based NO-releasing materials	Alginate is biodegradable and biocompatible; NO-releasing moieties easily introduced via abundant hydroxyl and carboxylic acid groups; NO release easily tunable by modifying high/low molecular weight alginate used	
NO-releasing cyclodextrins	Hydrophobic central cavity and hydrophilic exterior could enable delivery of hydrophobic antimicrobial compounds along with NO release	
NO-releasing silica nanoparticles	Innate antimicrobial activity of nanoparticles. Physiochemical properties, such as shape, sizes, and surface charge can be easily modified to improve NO delivery and bacteria eradication	Cytotoxicity reported in some designs
NO-releasing polymeric nanoparticles	Specificity and controlled release of NO can be achieved by incorporating photo-responsive groups and surface-charge switchable components; Able to co-deliver antibiotic with NO release to enhance bacterial or biofilm eradication; Other properties, e.g., magnetic field responsive NO-NP, may also be obtained	
NO-releasing dendrimers	High NO payloads within a single framework; Polymerization of antibiotics enable simultaneous delivery of NO with antibiotic and improve bacteria and biofilm eradication	Cytotoxicity may be associated with higher generation dendrimers and certain chemical modifications/ dendrimers
NO-releasing gel, polymer, and coatings	NO-releasing surfaces used in blood contacting medical devices may be designed to generate an NO flux representative of endothelial cells; Additional coating along with NO release can extent the anti-fouling lifespan of the material	Leaching of NO may occur depending on the design

## Abbreviations

Ag NPs	Silver nanoparticles
C3D	Cephalosporin-linked diazeniumdiolate NO-donor prodrugs
CD	Cyclodextrins
Ce6	Chlorin e6
CF	Cystic fibrosis
COS-NO	Chitosan oligosaccharides
CS/NO	NO-releasing chitosan
ED	1,2 -epoxy-9-decene
EPS	Extracellular polysaccharides
GSH	Gluthathione
GSNO	*S*-Nitrosoglutathione
ICG	Indocyanine green
IONPs	Iron oxide NPs
ISMN	Isosorbide mononitrate
LMW	Low molecular weight
MDR	Multidrug resistant
MPs	Microparticles
MW	Molecular weight
NIR	Near infrared
NO	Nitric oxide
NONOate	Diazeniumdiolates
NO-NPs	NO-releasing nanoparticles
NOS	NO synthase
NPs	Nanoparticles
PAMAM	Poly(amidoamine)
PDT	Photodynamic therapy
PEG	Polyethylene glycol
PGLA	Poly(lactic-co-glycolic acid)
PGMA	Poly(glycidyl methacrylate)
PKA	Poly kanamycin
PO	Propylene oxide
POEGMA	Poly (oligoethylene glycol methacrylate)
Poly(HEMA)	poly(hydroxyethyl methacrylate)
Poly(SMBA)	poly(sulfobetaine methacrylate)
PPI	Poly(propylene imine)
PTFE	Polytetrafluoroethylene
PTT	Photothermal therapy
PU	Polyurethane
PVBA	Poly (vinylbenzaldehyde)
QS	Quorum sensing
RNI	Reactive nitrogen intermediates
RNS	Reactive nitrogen species
ROI	Reactive oxygen intermediates
ROS	Reactive oxygen species
RSNO	*S*-nitrosothiols
SNAC	*S*-nitroso-N-acetylcysteine
SNAP	*S*-nitroso-N-acetylpenicillamine
SNP	Sodium nitroprusside
SWF	Simulated wound fluid
